# The mechanism of sesame resistance against *Macrophomina phaseolina* was revealed via a comparison of transcriptomes of resistant and susceptible sesame genotypes

**DOI:** 10.1186/s12870-021-02927-5

**Published:** 2021-03-29

**Authors:** Wenqing Yan, Yunxia Ni, Xintao Liu, Hui Zhao, Yanhua Chen, Min Jia, Mingming Liu, Hongyan Liu, Baoming Tian

**Affiliations:** 1 Institute of Plant Protection, Henan Academy of Agricultural Sciences, Postgraduate T&R Base of Zhengzhou University, Key Laboratory of Integrated Pest Management on Crops in Southern Region of North China, Henan Key Laboratory of Crop Pest Control, Zhengzhou, 450002 Henan China; 2 School of Agricultural Sciences, Zhengzhou University, Zhengzhou, 450001 Henan China

**Keywords:** *Macrophomina phaseolina*, *Sesamum indicum*, Transcriptome, Disease resistance, Molecular mechanism

## Abstract

**Background:**

Sesame *(Sesamum indicum)* charcoal rot, a destructive fungal disease caused by *Macrophomina phaseolina* (Tassi) Goid (MP), is a great threat to the yield and quality of sesame. However, there is a lack of information about the gene-for-gene relationship between sesame and MP, and the molecular mechanism behind the interaction is not yet clear. The aim of this study was to interpret the molecular mechanism of sesame resistance against MP in disease-resistant (DR) and disease-susceptible (DS) genotypes based on transcriptomics. This is the first report of the interaction between sesame and MP using this method.

**Results:**

A set of core genes that response to MP were revealed by comparative transcriptomics and they were preferentially associated with GO terms such as ribosome-related processes, fruit ripening and regulation of jasmonic acid mediated signalling pathway. It is also exhibited that translational mechanism and transcriptional mechanism could co-activate in DR so that it can initiate the immunity to MP more rapidly. According to weighted gene co-expression network analysis (WGCNA) of differentially expressed gene sets between two genotypes, we found that leucine-rich repeat receptor-like kinase (LRR-RLK) proteins may assume an important job in sesame resistance against MP. Notably, compared with DS, most key genes were induced in DR such as pattern recognition receptors (PRRs) and resistance genes, indicating that DR initiated stronger pattern-triggered immunity (PTI) and effector-triggered immunity (ETI). Finally, the study showed that JA/ET and SA signalling pathways all play an important role in sesame resistance to MP.

**Conclusions:**

The defence response to MP of sesame, a complex bioprocess involving many phytohormones and disease resistance-related genes, was illustrated at the transcriptional level in our investigation. The findings shed more light on further understanding of different responses to MP in resistant and susceptible sesame.

**Supplementary Information:**

The online version contains supplementary material available at 10.1186/s12870-021-02927-5.

## Background

Plants have evolved complex signalling systems and molecular mechanisms to cope with multifarious biotic and abiotic stresses in a constantly changing environment [1]. In 2006, the concept of plant innate immunity was first proposed. Pattern-triggered immunity (PTI) and effector-triggered immunity (ETI) are two vital mechanisms in the long course of coevolution of plant and pathogen interactions [2]. Although PTI is feeble, it is essential for plants and is the first line of defence against pathogens [3]. PTI can be triggered by pathogen-associated molecular patterns (PAMPs), followed by thickening of cell wall, lignification of cell wall, production of phytoalexin and induction of the expression of PR genes. However, some pathogens can restrain and break through the defence of PTI by secreting effectors into plants. Plants have developed a reconnaissance mechanism to perceive and recognize these effectors, which leads to ETI. Both PTI and ETI are engaged in the early defence response of plants. They perform comparative functions and early induction of defence signalling transduction and downstream molecular network responses can also be observed at the physiological level, such as the burst of reactive oxygen species (ROS), the activation of mitogen activated protein kinase (MAPK) pathway and amassing of callose [4]. The production of reactive oxygen intermediates, particularly the burst of superoxide anion radicals and the accumulation of hydrogen peroxide, is considered to be an early defence response of plants to external pathogens and is a necessary autoimmune reaction process of plants [5]. ROS, including O^2−^, H_2_O_2_ and HO^−^, predominantly collect in chloroplasts and mitochondria, and they can cause oxidative damage to lipids, proteins, nucleic acids and photosynthetic devices. To reduce oxidative harm, plants produce different types of antioxidant enzymes such as superoxidase dismutase (SOD), catalase (CAT) and peroxidase (POD) to scavenge ROS to enhance their disease resistance. Simultaneously, the process of decomposing H_2_O_2_ by POD can likewise produce toxic substances to ward off invasive microorganisms and inhibit the proliferation and diffusion of pathogens [6]. ROS are considered to be an essential signalling component in plant defence [7, 8].

Basic helix-loop-helix (bHLH) proteins belong to the TF superfamily and are widely distributed in eukaryotes. Members of the bHLH superfamily generally contain two highly conserved and functionally different domains: the N-terminal basic region that binds to DNA [9], which mainly recognizes the E-box and G-box [10], and the C-terminal HLH domain, which depends on the interaction of hydrophobic amino acids to form autodiploid or allodiploid of two HLH proteins and regulates the expression of downstream target genes [11, 12]. bHLHs often cooperate with members of other TF families to regulate and induce the biosynthesis of an assortment of secondary metabolites, such as terpenoids, alkaloids, phenylpropanoid, and anthocyanins and so on, which assume a significant job in regulating the interaction between plants and the environment [13, 14]. Presently, an ever-increasing number of studies have demonstrated that bHLHs are related to biotic or abiotic stress reactions in various plants [15–17].

Sesame, a member of the Pedaliaceae family, is one of the most advantageous and nutritious oil crops with an oil content of 50–60% and a protein content of 20–30%. Furthermore, it is rich in unsaturated fats (approximately 85%) and natural antioxidants such as sesamol, tocopherol and nutrient E [18, 19]. These antioxidants have significant health-promoting effects, such as reducing cholesterol and hypertension, reducing the incidence of some cancers and providing neuroprotective effects against hypoxia. Subsequently, the worldwide demand for sesame has continuously expanded as of late. However, in China, sesame is vulnerable to a variety of pathogens, which are the leading causes for the low and unstable yield of sesame. In addition, basic researches on sesame are still scarce compared with other crops, which is also one of the reasons for the low yield of sesame. It is necessary to study the basic genetics and molecular biology of sesame to improve the resistance of sesame to biotic stress.

Sesame charcoal rot generally occurs at the end of the flowering stage and before the ripening stage in sesame, with the disease spots beginning to appear and spread from the root or stem under hot and dry weather or high environmental stress. This is caused by the seed- and soil-borne fungus MP, which is highly contagious and can infect in excess of 500 species of plants. Charcoal rot usually diminishes sesame production of 10–15% or even over 80% in serious cases. Furthermore, it will likewise impact the quality of sesame by diminishing the oil content of sesame seeds of 4.2–16.2% [20]. Hence, revealing the resistance mechanism of sesame, screening for the resistance genes in sesame and cultivating resistant varieties are helpful for alleviating the loss of yield. Unfortunately, the genetic improvement of sesame is proceeding slowly due to the lack of a known molecular mechanism and information regarding the gene–for–gene relationships in the interaction between sesame and MP. With the publication of sesame genomes [21, 22], researches on sesame have become increasingly active. Hitherto, an investigation of the transcriptomes involved in the interaction between sesame and MP has not yet been published.

Consequently, this study preliminarily explored the molecular mechanism of sesame resistance to MP by comparing and analysing the transcriptome data of a sesame resistant genotype DR and a susceptible genotype DS inoculated with MP, which will provide a fundamental theoretical research for the genetic improvement of sesame.

## Results

### Root phenotypes of DS and DR post inoculation by MP

**Fig. 1:**
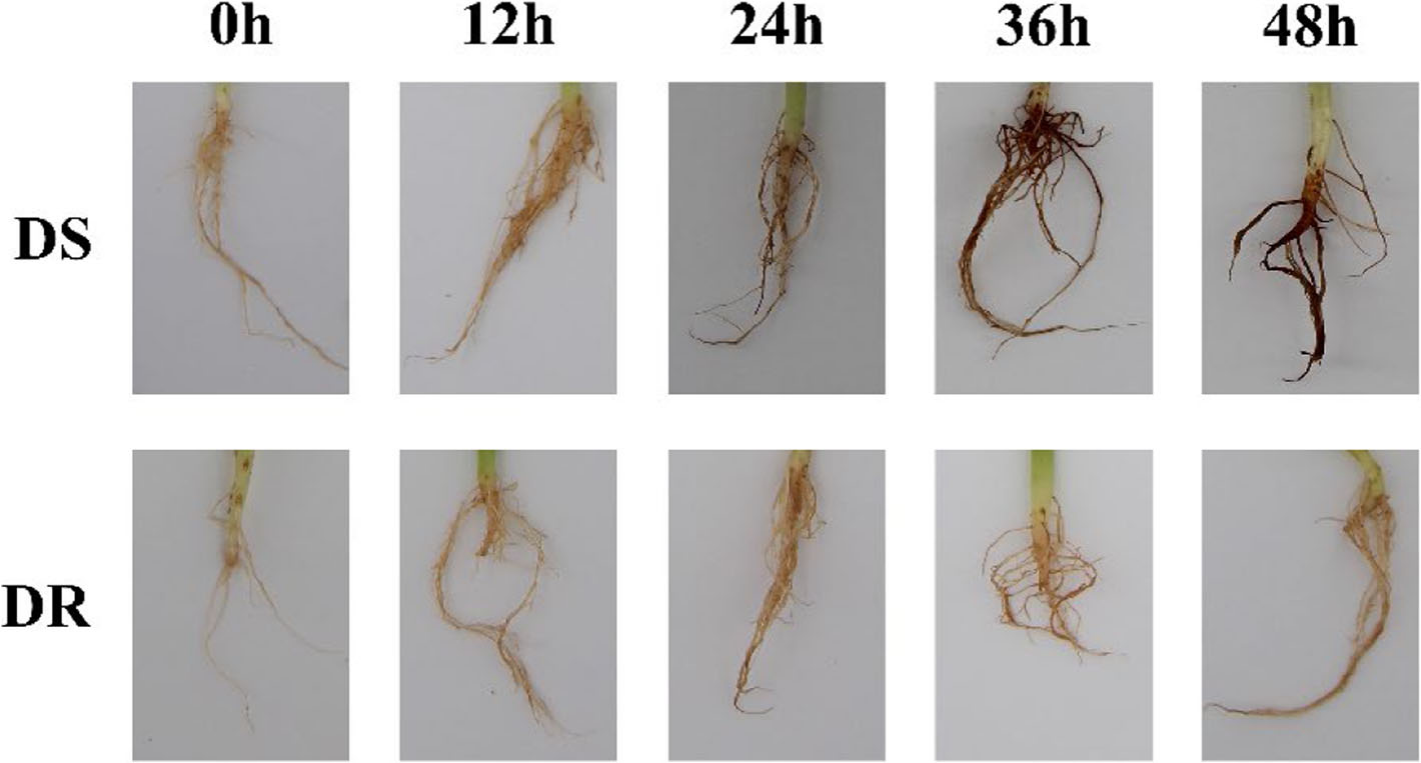
Root phenotypes of DS and DR post innoculation by MP

According to the results of infection phenotype of sesame roots, we found that there was a difference between DR and DS post-inoculation by MP (Fig. 1). The roots of DS and DR showed no significant difference before inoculation. At 12 h post-inoculation (HPI), sporadic black spots were observed in the roots of DS, while no obvious symptoms in DR. At 24 HPI, the black spots in DS were increasing significantly and necrosis can be seen in a part of the roots. However, the black spots just appeared in the roots in DR at this moment. With the passage of time (36 HPI and 48 HPI), the black spots in DS gradually spread to the whole root and the necrotic area further expanded, while in DR, the black spots didn’t change significantly, and there was no obvious necrosis in the root within 48 h. It further indicates that DS is susceptible to MP, while DR is highly resistant to MP.

### Illumina sequencing and alignment to the genome

Plant at five time points post-inoculation (0 HPI, 12 HPI, 24 HPI, 36 HPI, and 48 HPI) and their biological replicates were sequenced and 30 transcriptomes were obtained. The evaluation of the sequencing quality indicated that the sequencing results of all samples were excellent, the base distribution was balanced and the mean Q value was approximately 36. More than 2.4 billion raw reads were generated from 30 libraries, and then approximately 2.337 billion clean reads (clean ratio > 95.69%) were obtained for subsequent analysis after removing the adapter sequences, low-quality reads and rRNA sequences. On average, 95.89% of the reads could be mapped to the reference genome of sesame and most of them (88.28%) could be mapped to the coding regions (Additional file: Table S1).

**Fig. 2:**
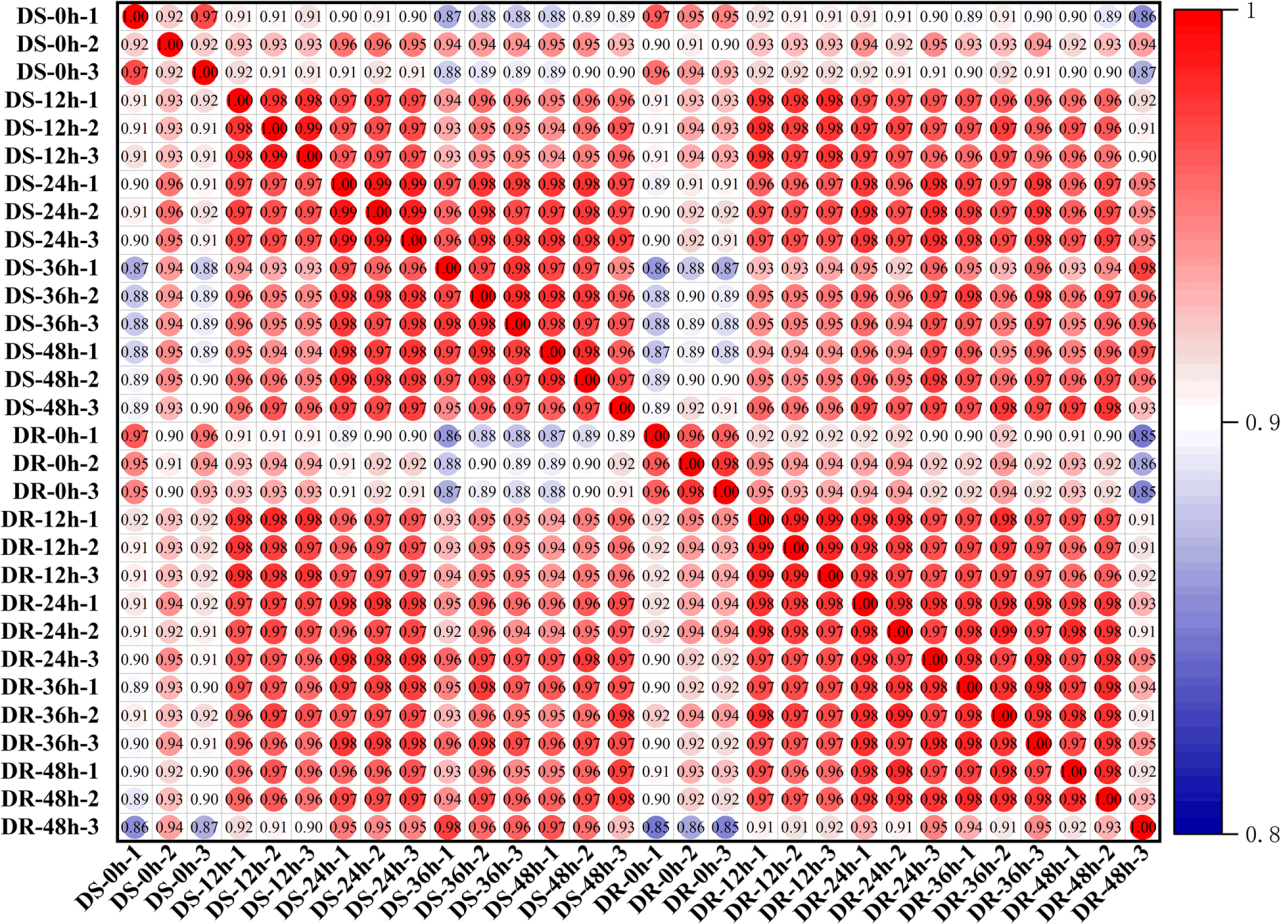
Pearson correlation coefficients of all 30 samples. The expression level of each gene for each pair of samples was used to calculate the Pearson correlation coefficients

The relationships among the samples were checked through the Pearson correlation coefficients between samples (Fig. 2) and principal component analyses (PCA) (Additional file: Figure S1). We selected samples with a high correlation between biological replicates (R^2^ > 0.96). Furthermore, we can also see that the two samples DS 0 h-2 and DR 48 h-3 were serious outliers based on PCA. Thus, DS 0 h-2 and DR 48 h-3 were excluded from the following analysis. PCA also showed that there was a great difference between the control (0 HPI) and treated groups, which indicated that MP induced many different changes in the sesame transcriptomes, and there may be some resistance-related genes in the interaction between sesame and MP.

### Core gene sets in response to MP

In order to apprehend the overall transcriptome changes in the interaction between sesame and MP in the two genotypes, the genes with FPKM values greater than 0.1 were regarded as expressed genes. A total of 22,049, 22,114, 21,961, 21,712 and 22,032 genes were detected in DS, while 22,514, 22,036, 22,032, 22,049 and 22,100 genes were detected in DR at 0 HPI, 12 HPI, 24 HPI, 36 HPI, and 48 HPI, respectively. The expression of 23,042 and 23,217 genes was also observed at all time points in DS and DR, respectively (Additional file: Figure S2A). There are 22,761 genes (96.9%) expressed in both DS and DR, 281 genes (1.2%) were specifically expressed in DS and 456 (1.9%) in DR (Additional file: Figure S2B).

To investigate genes involved in the response to MP in sesame, differentially expressed genes (DEGs) were identified under the standard of false discovery rate (FDR) < 0.01 and |log2-fold change| > 1. As shown in additional file: Figure S3A, 3607, 3876, 3336 and 3359 DEGs were significantly up-regulated and 2839, 3684, 4329 and 2956 DEGs down-regulated in DS, while 2304, 2410, 2485,2600 DEGs in DR were significantly up-regulated and 2803,2703,3091 and 2394 DEGs down-regulated at four time points post-inoculation, respectively. It follows that the overall DEGs (4994–5576 DEGs) of DR ware fewer than those of DS (6315–7665 DEGs) within 48 h post-inoculation, and the number of DEGs responding to stress in DR was significantly fewer than that in DS at each time point. This indicated that the injury caused by MP in DR was likely to be much less than that in DS, which may change the transcriptome expression profile to a smaller extent and permit the plant to cope with the stress more easily.

Further overlap analysis of up- and down-regulated DEGs at four time points of DS and DR showed that 1977 and 1320 co-up-regulated genes and 1791 and 1357 co-down-regulated genes were identified in DS and DR respectively. To identify the core gene sets in response to MP, we compared the overlap DEGs between DS and DR and found that there are 867 up-regulated DEGs and 721 down-regulated DEGs overlapped between the two genotypes (Additional file: Figure S3B).

**Fig. 3:**
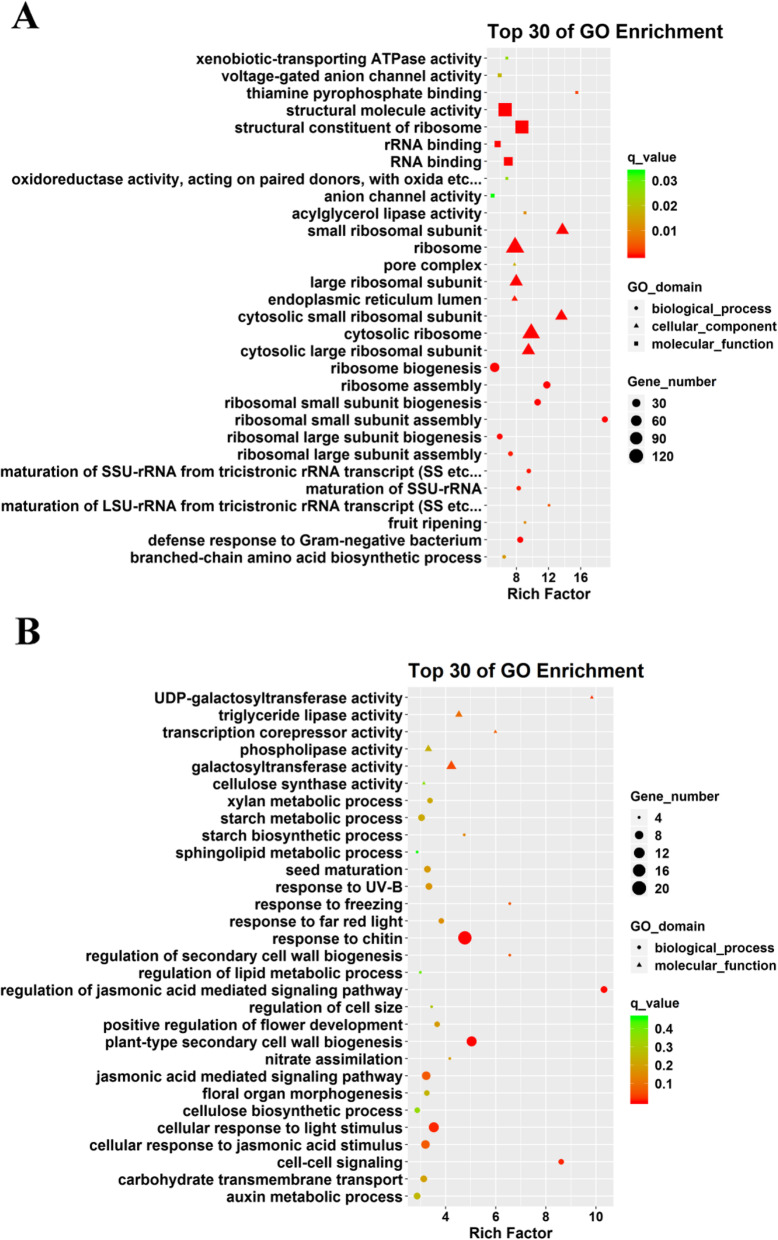
Top 30 GO term enriched functional categories of co-up-regulated (**a**) and co-down-regulated (**b**) DEGs in the two genotypes

The enrichment of GO terms of the core gene sets above was analysed to study the potential function of genes in response to MP. The 867 up-regulated DEGs were mainly enriched in ribosome-related processes, followed by thiamine pyrophosphate binding, maturation of LSU-rRNA from tricistronic rRNA transcript (SSU-rRNA, 5.8S rRNA, LSU-rRNA), fruit ripening, acylglycerol lipase activity and defence response to gram-negative bacterium (Fig. 3a). Likewise, the main term with the highest enrichment of 721 down-regulated DEGs are regulation of jasmonic acid mediated signalling pathway, UDP-galactosyltransferase activity, cell-cell signalling, response to freezing and regulation of secondary cell wall biogenesis (Fig. 3b).

### DEGs up-regulated uniquely in DR

To study the functional specificity of disease resistance in DR, up-regulated DEGs observed specifically in DR compared with DS were explored over time. At 12 HPI, 733 DEGs were enriched in the process of ribosome synthesis and assembly, maturation of SSU-rRNA from tricistronic rRNA transcript (SSU-rRNA, 5.8S rRNA, LSU-rRNA) and cytoplasmic translation (Additional file: Figure S4A), indicating that DR may respond more quickly than DS and prepare for the translation of resistance-related proteins at the level of transcription and translation during the initial stage of stress. At 24 HPI, 488 DEGs were enriched in GO terms such as ligand-gated ion channel activity, cellular response to hypoxia, oxidoreductase activity, systemic acquired resistance, and positive regulation of defence response (Additional file: Figure S4B), which reveals that DR has made a series of responses to infection stress, such as the production of peroxidase and activation of the systemic acquired resistance process and defence response, illustrating that DR can arrange the defence system more quickly and effectively to resist MP. When the stress was more severe (36 HPI), a total of 737 DEGs were specifically up-regulated in DR, which were mainly enriched in ribosome-related processes, followed by phloem transport, nucleoside, nucleobase transport, nucleobase transmembrane transporter activity, cytoplasmic translation and hydrogen peroxide catabolic process (Additional file: Figure S4C). At 48 HPI, GO enrichment indicated that 750 DEGs were involved in ribosome related pathways, cytoplasmic translation, RNA binding, beta-glucosidase activity, ligand-gated ion channel activity and monoterpenoid biosynthetic process (Additional file: Figure S4D). This result indicated that DR continuously transcribes, translates and transports disease resistance-related proteins and secondary metabolites such as monoterpenes to address stress. Overall, the rapid stress responses and the activation of specific disease-related pathways of DR might lead to its high resistance.

### DEGs between DS and DR

**Fig. 4:**
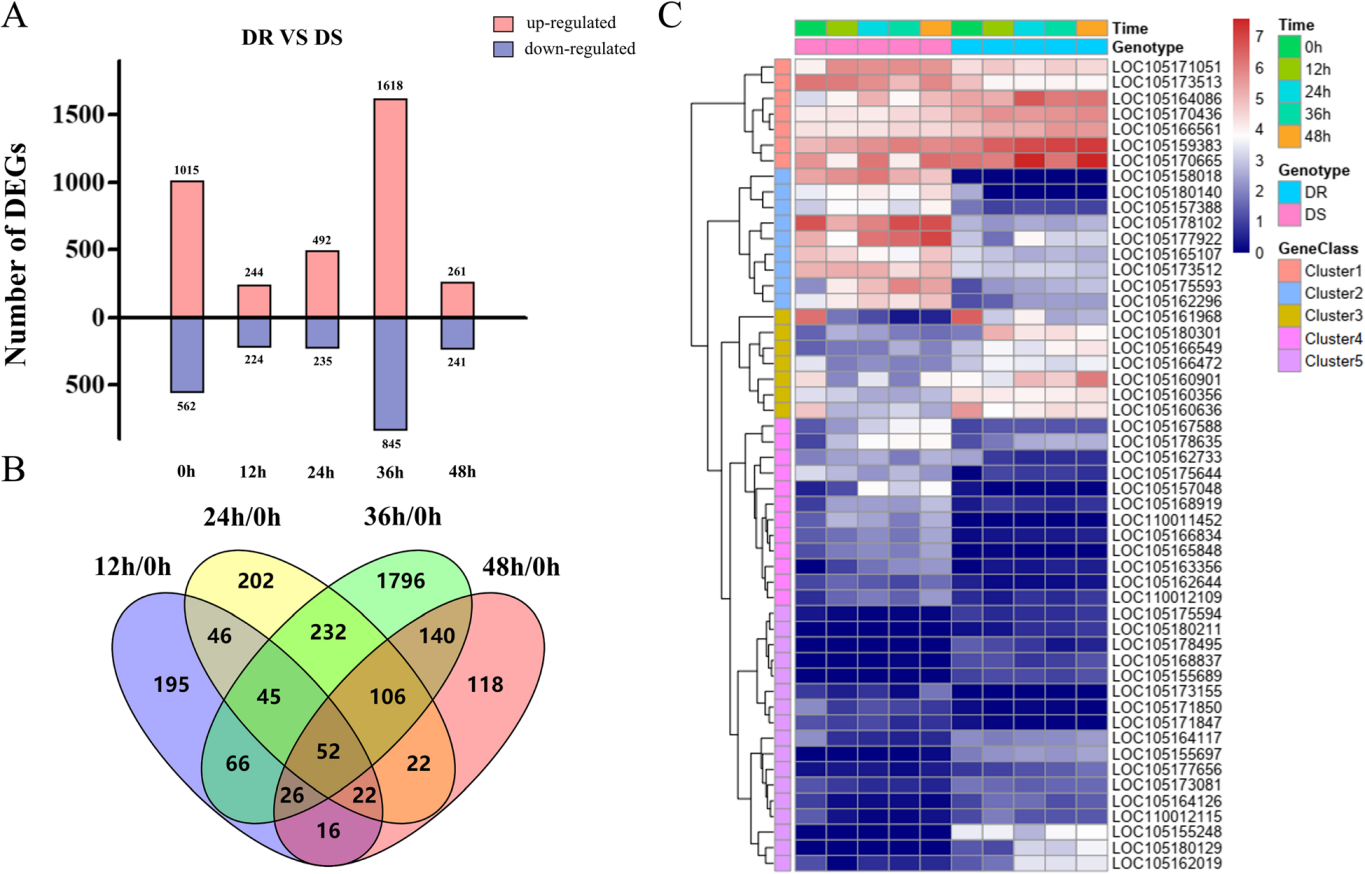
DEGs compared between DR and DS post-inoculation. **a**. Numbers of DEGs between DR and DS during MP stress. **b**. Common and unique DEGs between DR and DS post-inoculation. **c**. Expression patterns of 52 common DEGs between DR and DS post-inoculation. The value of gene expression is shown as log_2_(FPKM+ 1)

Moreover, DEGs were compared between DR and DS to filter the genes with a high correlation with disease resistance in sesame. Under normal growth conditions (0 HPI), 1577 DEGs were observed between DR and DS (Fig. 4a). GO enrichment analysis showed that these genes were enriched in condensin complex, mitotic chromosome condensation, DNA primase activity, chromosome condensation, DNA unwinding involved in DNA replication, FMN reductase activity and response to anoxia (Additional file: Figure S5).

Then, based on overlapped analysis, a total of 52 genes that were significantly differentially expressed between the two genotypes at all four time-points were obtained (Fig. 4b). Then, they were classified into 5 clusters exhibiting different functions (Fig. 4c). These 52 genes were most enriched in GO terms such as response to abscisic acid, cell wall, hormone-mediated signalling pathway, response to hormone, cell-cell junction, defence response and signal transduction (Additional file: Figure S6), most of which are known to be associated with plant stress. This further implies that these genes are crucial candidate genes inducing higher resistance in DR than DS. Among them, 20 of these genes exhibited significant differences in expression even under normal conditions between the two genotypes.

It is noteworthy that most DEGs were found in the two sesame genotypes at 36 HPI, as well as between the two genotypes. Furthermore, many DEGs in the KEGG pathways related to biological stress were significantly enriched at 36 HPI in DR VS DS, including “plant hormone signal transduction” (35 DEGs), “plant-pathogen interaction” (15 DEGs), “brassinosteroid biosynthesis” (2 DEGs) and “ diterpenoid biosynthesis “ (12 DEGs). However, DEGs in these key signalling pathways were not obvious at other time points, indicating that 36 HPI may be an important period in the disease resistance of sesame.

### DEGs involved in key pathways at 36 HPI

From the perspective of KEGG pathways, “plant-pathogen interaction” and “plant hormone signal transduction” are the key pathways in plant resistance. Therefore, the two main pathways in sesame were analysed at the important time of 36 HPI.

The genes involved in the “plant-pathogen interaction” pathway were identified based on KEGG pathway assignment. The results showed that 48 and 72 DEGs were identified in DR and DS, respectively, and most of these genes were down-regulated in both genotypes. In DR, the expression of PR1 (pathogenesis-related protein 1), HSP90 (heat shock protein 90 kDa beta), MAP 2 K1 (mitogen-activated protein kinase kinase 1) and RPM1 (disease resistance protein RPM1) increased, while the expression of WRKY22, WRKY29, WRKY33, Rboh (respiratory burst oxidase), FLS2 (LRR receptor-like serine/threonine-protein kinase FLS2) and CDPK (calcium-dependent protein kinase) decreased. In DS, the expression of BAK1 (brassinosteroid insensitive 1-associated receptor kinase 1), HSP90, MAP 2 K1 and Pti1 (pto-interacting protein 1) genes were up-regulated, while FLS2, MEKK1 (mitogen-activated protein kinase kinase kinase 1), NHO1 (glycerol kinase), Rboh, RPS2 (disease resistance protein RPS2), WRKY22, WRKY29 and WRKY33 were down-regulated. Furthermore, in the “plant-pathogen interaction” pathway, up-regulated genes such as HSP90 were more involved in DR than in DS. For the genes that were detected only in DS, the expression of BAK1 and Pti1 increased while the expression of MEKK1 and NHO1 decreased (Additional file: Table S2).

Similarly, in the “plant hormone signal transduction” pathway, there were more DEGs involved in DS (113) than in DR (76). Most of these genes participate in auxin (AUX), abscisic acid (ABA) and ethylene (ET) biosynthesis. In addition, genes connected to CRE1 (Arabidopsis histidine kinase 2/3/4), B-ARR (two-component response regulator ARR-B family), SnRK2 (serine/threonine-protein kinase SRK2), EIN2 (ethylene-insensitive protein 2), BZR1_2 (brassinosteroid resistant 1/2) and BSK (BR-signalling kinase) specifically expressed in DS were all down-regulated while those related to BAK1 and BKI1 were all up-regulated (Additional file: Table S2).

In the “ plant-pathogen interaction” pathway, in comparison with DS, the expression levels of 2 CDPKs, 3 disease resistance protein RPM1, 1 LRR receptor-like serine/threonine-protein kinase FLS2, 2 CML genes encoding calcium-binding protein and 1 Rboh gene in DR were significantly up-regulated. These genes can induce PTI in plants by identifying Ca^2+^ signals and then activate hypersensitive responses and cell wall reinforcement to prevent the spread of pathogens. The expression levels of other genes such as WRKY22 were significantly up-regulated, which can induce resistance by generating downstream defence genes. On the other hand, a total of 35 genes were differentially expressed in “plant hormone signal transduction” pathway, with 11 DEGs up-regulated in connection with auxin, including 2 IAA (auxin-responsive proteins), 2 AUX1 (auxin influx carriers), 2 auxin-responsive GH3, 1 ARF (auxin response factor) and 4 SAUR proteins. The other up-regulated genes were 2 genes encoding ethylene-responsive transcription factor (ERF1) involved in ethylene response, 2 transcription factors TGA and 1 regulatory protein NPR1 related to SA biosynthesis, 2 cyclin D3 (CYCD3) involved in the brassinosteroid pathway, 1 DELLA protein involved in gibberellin biosynthesis and 2 genes encoding two-component response regulator related to the cytokinin synthesis pathway (Table 1).

**Table 1:** DEGs between DR and DS in plant-pathogen interaction and plant hormone signal transduction pathways at 36 HPI

Gene ID	Gene symbol	LOG2(FC)	pathway
LOC105166461	CALM	1.67	Plant-pathogen interaction
LOC105171604	CALM	1.66	Plant-pathogen interaction
LOC105165972	CDPK	1.16	Plant-pathogen interaction
LOC105159534	CDPK	1.77	Plant-pathogen interaction
LOC105165460	Rboh	1.19	Plant-pathogen interaction
LOC105171969	RPM1	1.57	Plant-pathogen interaction
LOC105157411	RPM1	2.38	Plant-pathogen interaction
LOC105160106	RPM1	1.77	Plant-pathogen interaction
LOC105173039	WRKY22	1.52	Plant-pathogen interaction
LOC105165316	CALM	−1.41	Plant-pathogen interaction
LOC105180110	CALM	−1.20	Plant-pathogen interaction
LOC105173088	CNGC	−1.28	Plant-pathogen interaction
LOC105164060	HSP90	−1.15	Plant-pathogen interaction
LOC105172653	PR1	−2.70	Plant-pathogen interaction
LOC105175642	ARF	1.06	Plant hormone signal transduction
LOC105174548	AUX1	1.41	Plant hormone signal transduction
LOC105160898	AUX1	1.22	Plant hormone signal transduction
LOC105162066	B-ARR	3.56	Plant hormone signal transduction
LOC105156670	B-ARR	1.25	Plant hormone signal transduction
LOC105159150	CYCD3	1.23	Plant hormone signal transduction
LOC105169513	CYCD3	1.72	Plant hormone signal transduction
LOC105178897	DELLA	5.08	Plant hormone signal transduction
LOC105161291	ERF1	4.08	Plant hormone signal transduction
LOC105167788	ERF1	3.12	Plant hormone signal transduction
LOC105176748	GH3	1.70	Plant hormone signal transduction
LOC105174793	GH3	1.22	Plant hormone signal transduction
LOC105171637	IAA	1.27	Plant hormone signal transduction
LOC105171811	IAA	1.18	Plant hormone signal transduction
LOC105159880	NPR1	1.87	Plant hormone signal transduction
LOC105156793	SAUR	2.17	Plant hormone signal transduction
LOC105155428	SAUR	2.33	Plant hormone signal transduction
LOC105174292	SAUR	3.22	Plant hormone signal transduction
LOC105166772	SAUR	1.55	Plant hormone signal transduction
LOC105171237	TGA	1.80	Plant hormone signal transduction
LOC105176696	TGA	2.09	Plant hormone signal transduction
LOC105156765	A-ARR	−2.35	Plant hormone signal transduction
LOC105159175	BKI1	−1.44	Plant hormone signal transduction
LOC105177517	DELLA	−1.65	Plant hormone signal transduction
LOC105158452	ERF1	−1.08	Plant hormone signal transduction
LOC105164449	ERF1	−1.37	Plant hormone signal transduction
LOC105171710	ERF1	−1.09	Plant hormone signal transduction
LOC105155519	GID2	−1.02	Plant hormone signal transduction
LOC105168467	JAZ	−3.20	Plant hormone signal transduction
LOC105172653	PR1	−2.70	Plant hormone signal transduction
LOC105175593	ERF1	−3.45	Plant hormone signal transduction
LOC105171100	SAUR	−1.26	Plant hormone signal transduction
LOC105158157	SAUR	−1.55	Plant hormone signal transduction
LOC105173359	SAUR	−1.82	Plant hormone signal transduction
LOC105165572	TCH4	−2.08	Plant hormone signal transduction

### Analysis of hub genes by WGCNA between two sesame genotypes

**Fig. 5:**
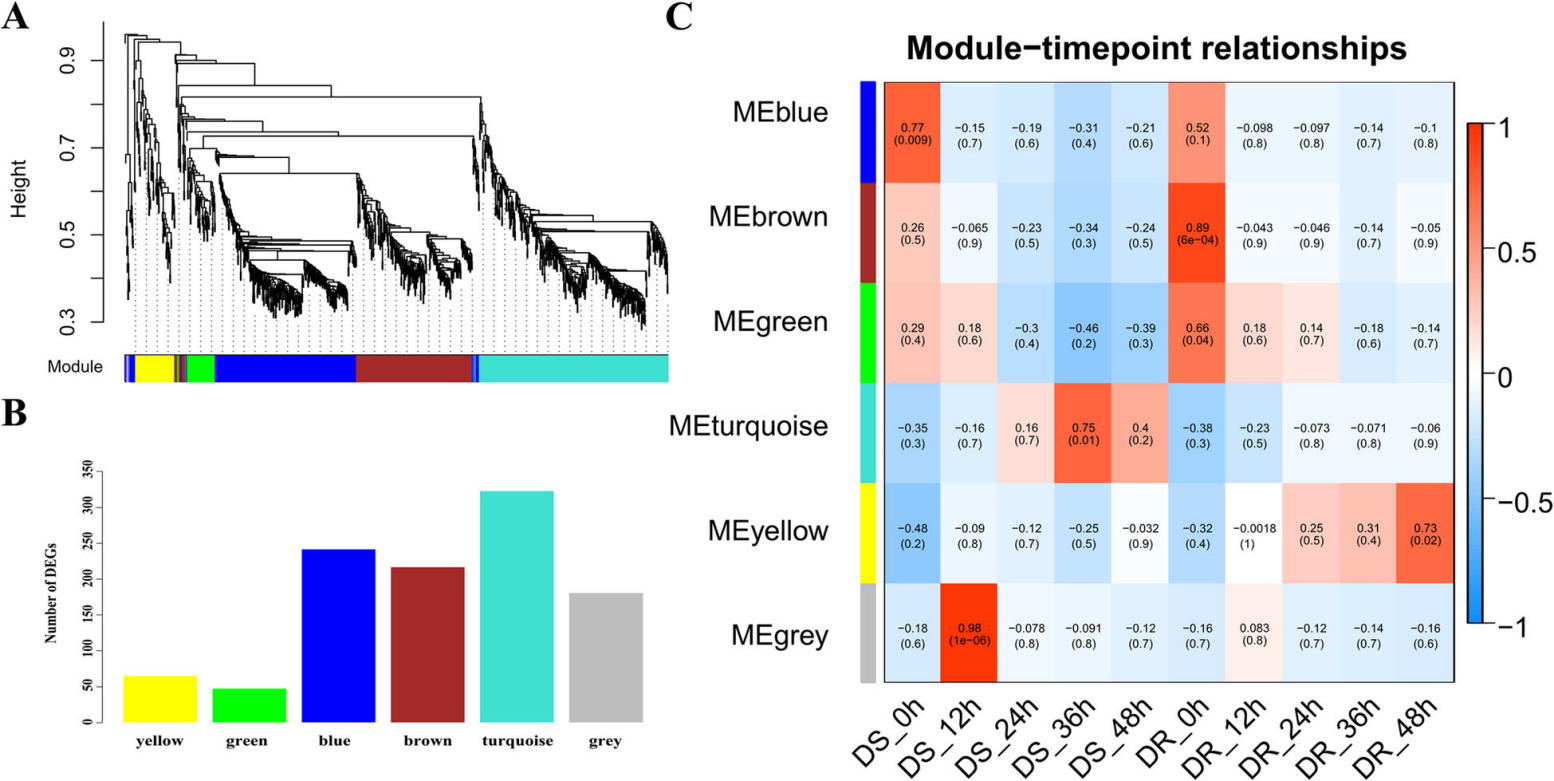
Detection of co-expressed modules of MP stress responsive genes according to WGCNA. **a**. Cluster Dendrogram of different genes in co-expression modules. **b**. Number of genes in different modules. **c**. Relationships between co-expressed modules and timepoints in DS and DR

All the genes (mean FPKM > 0.1) differentially expressed between DR and DS post-inoculation were further investigated by weighted gene co-expression network analysis (WGCNA). 1076 genes were divided into six co-expression modules named as blue, brown, green, turquoise, yellow and grey, containing 242, 217, 48, 323, 65 and 181 genes, respectively (Fig. 5a, b). The correlation between the detected modules and the time-points post-inoculation of resistant and susceptible genotypes showed that all the six modules differently response to MP stress. Among these modules, the genes in the turquoise module were negatively correlated with DR-36HPI, but positively correlated at DS-36HPI. Similarly, the genes in the yellow module were positively correlated with DR-36HPI, but negatively correlated at DS-36HPI (Fig. 5c).

**Fig. 6:**
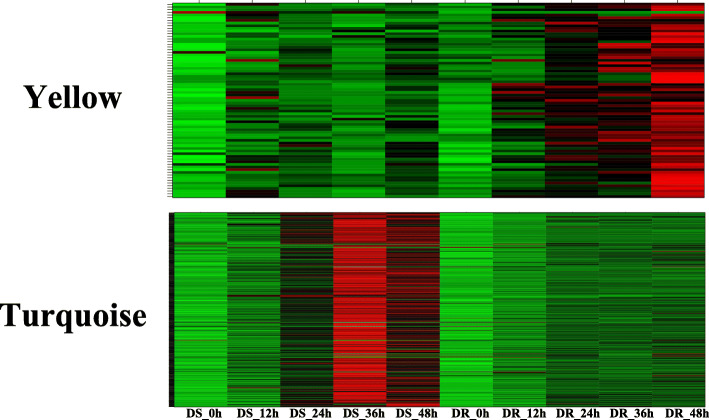
Heatmaps of gene expression patterns for yellow and turquoise modules

**Fig. 7:**
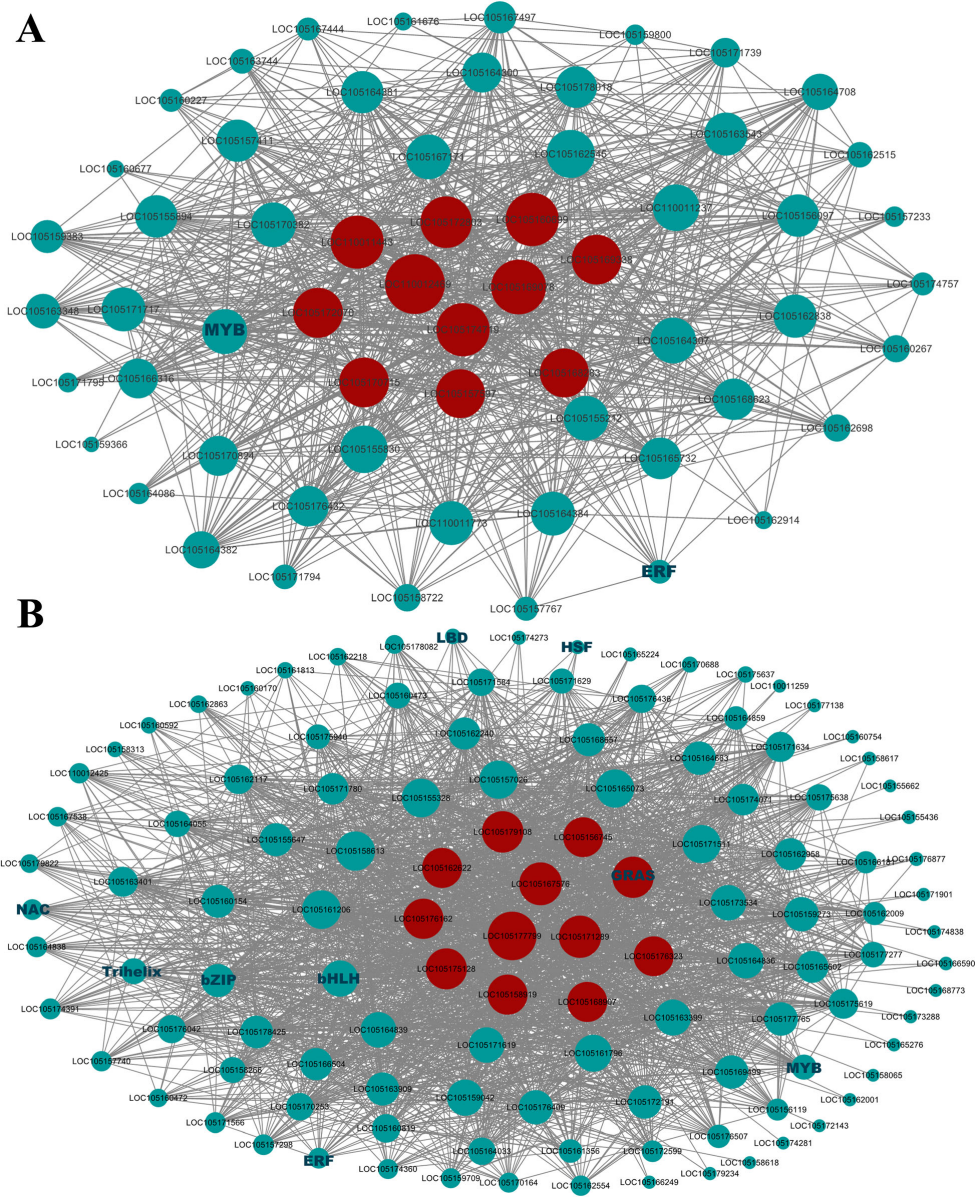
Co-expressed network analysis of yellow module (**a**) and turquoise module (**b**). The size of node circle is positively correlated with the number of genes it interacts. TFs are represented with darkgreen font and hub genes are showed as red nodes

The expression pattern of the genes in these two modules is shown in Fig. 6. This indicated that these two modules may contain resistant genes to defence MP, so we selected turquoise and yellow modules for gene co-expression network analysis to reveal hub genes during the interaction between sesame and MP. In gene co-expression networks, many genes only interact with a limited number of others while fewer gene sets (hub genes) interact with many others, there is no doubt hub genes in the networks play a core role. In order to understand the relationship between the genes within the modules, Cytoscape software was used to construct the gene networks of the yellow and turquoise modules (weight > 0.3 and the first 2000 edges). TFs are represented with darkgreen font and the size of node circle is positively correlated with the number of genes it interacts. Genes with biggest node sizes represent the hub genes and they are showed as red nodes (Fig. 7).

In yellow module, we observed several hub genes, including LOC105160699 (LRR receptor-like serine/threonine-protein kinase), LOC105172070 (probable LRR receptor-like serine/threonine-protein kinase), LOC110012469 (probable LRR receptor-like serine/threonine-protein kinase), LOC105170715 (aquaporin PIP2–7), LOC105157597 (probable 2-oxoglutarate-dependent dioxygenase), LOC105172803 (polygalacturonase), LOC105169338 (histone H3.2), LOC105168283 (2-phytyl-1,4-beta-naphthoquinone methyltransferase), LOC105169078 (protein DETOXIFICATION 43), LOC110011443 (NA) and LOC105174719 (NA). Similarly, The hub genes detected in turquoise module were LOC105161270 (GRAS), LOC105177799 (peroxidase 73 precursor), LOC105158919 (laccase-15-like), LOC105179108 (laccase-14-like), LOC105175128 (G-type lectin S-receptor-like serine/threonine-protein kinase), LOC105156745 (E3 ubiquitin-protein ligase RHA2B-like), LOC105171289 (ATP sulfurylase 1), LOC105168907 (linoleate 9S-lipoxygenase 5), LOC105167576 (VQ motif-containing protein 22), LOC105162622 (olee1-like protein), LOC105176162 (NA), LOC105176323 (NA). Furthermore, some key TFs like LOC105174354 (MYB) in yellow module and LOC105173824 (bHLH) and LOC105161270 (GRAS) in turquoise module were also detected, which may play vital regulation role in defence. These results suggest that the genes encoding LRR-RLK and laccase may play a major role in the sesame defence against MP. At the same time, the activity of peroxidase in sesame may contribute to the resistance of sesame. Notably, LOC105161270 (GRAS) is not only a hub gene in the turquoise module, but also a TF closely related to disease resistance in plants, indicating that it may be a crucial regulatory gene in the resistance to MP.

### TFs involved in sesame defence

**Fig. 8:**
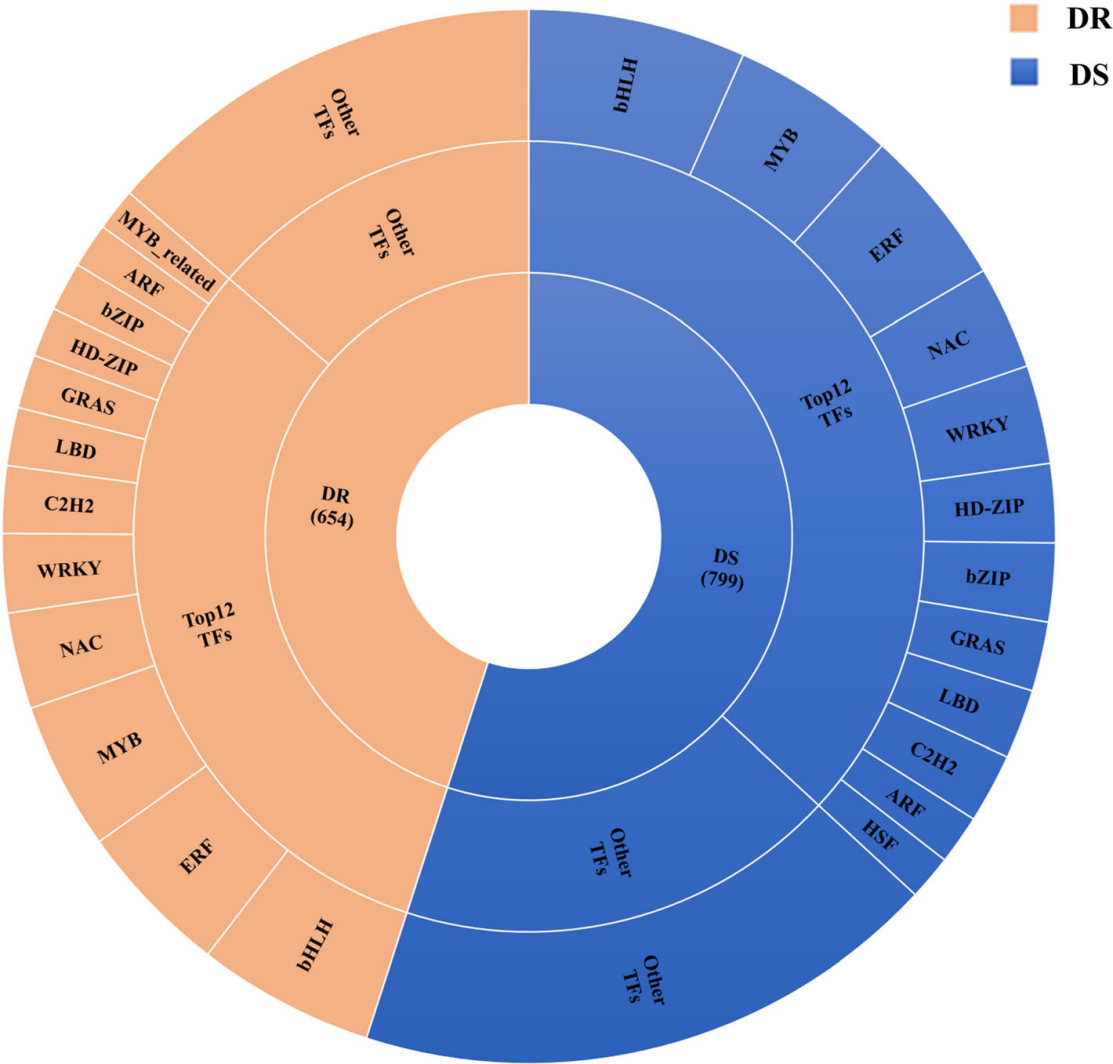
Overall TFs in DR and DS post-inoculation

To research the major TFs of sesame in the interaction between sesame and MP, we investigated the expression of all genes involved in transcriptional regulation. A total of 3904 TFs were identified in the DEGs in sesame, which were grouped into 49 gene families. In general, the number of transcription factors increased with the severity of stress (Additional file: Table S3). In DR, bHLH gene family, the most abundant and active TF family, was significantly more represented than other TF families, followed by ERF, MYB, NAC, WRKY, C_2_H_2_, LBD, GRAS, HD-ZIP, bZIP, ARF, MYB_related and other transcription factor families. Additionally, many DEGs were members of the TF families bHLH, MYB, ERF, NAC, WRKY, HD-ZIP, bZIP, GRAS, LBD, C_2_H_2_, ARF and HSF in DS (Fig. 8). In this research, it was discovered that the bHLH transcription factor family was the most abundant transcription factor in the interaction between sesame and MP, indicating that bHLH proteins may play a vital role in sesame charcoal rot resistance.

### Real-time quantitative PCR

**Fig. 9:**
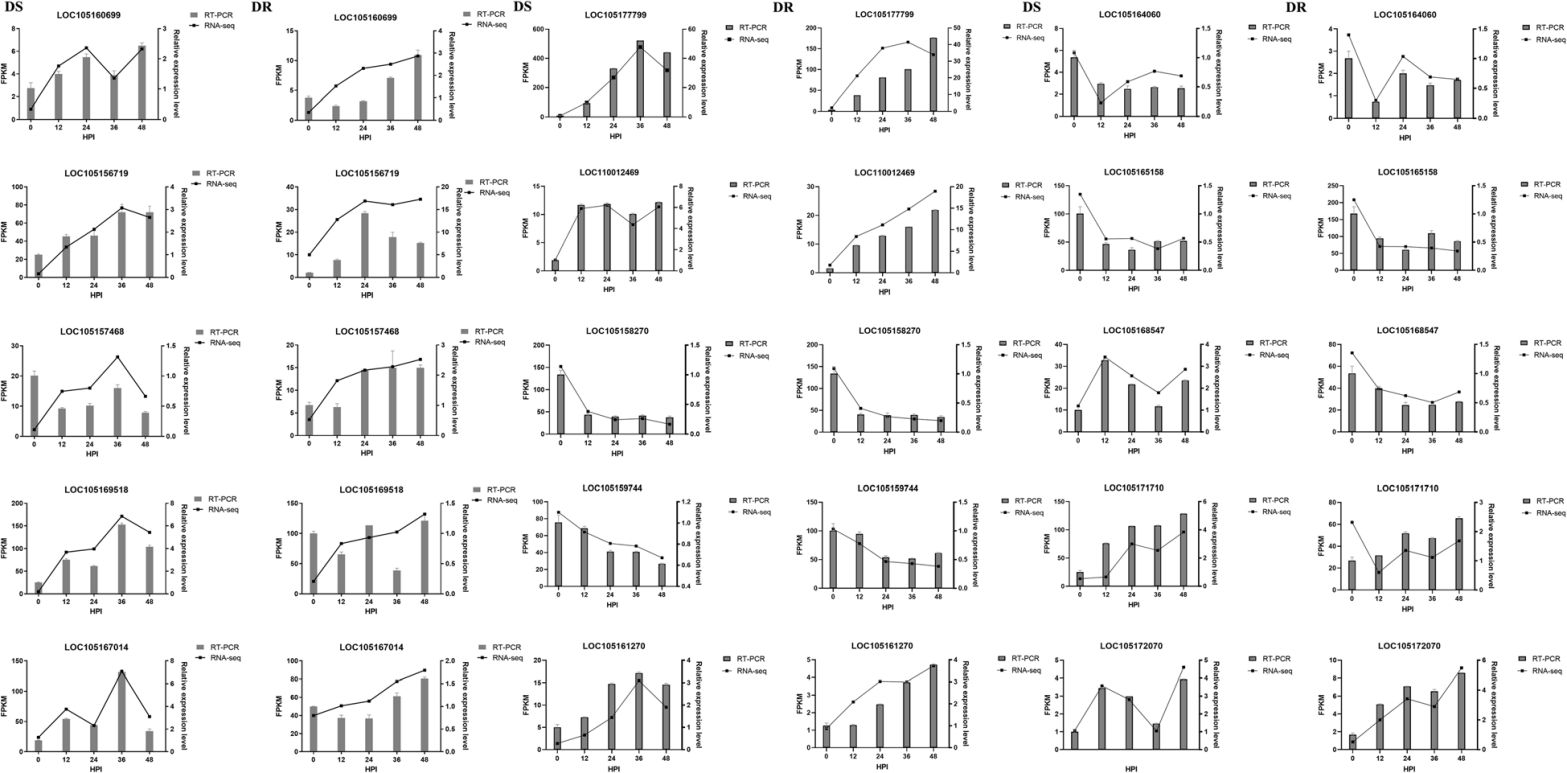
Quantitative RT-PCR validation of genes in DS and DR

15 genes in two genotypes that responded to MP were selected to confirm the RNA-seq results, including 8 genes involved in resistant to MP and 7 genes in phytohormone signalling pathway (Additional file: Table S4). The results show that the real-time quantitative PCR and RNA-seq are consistent with the overall expression trend (Fig. 9), indicating that the RNA-seq used in this study showed a high degree of reliability.

## Discussion

### Comparative transcriptome analysis

Plants are generally subjected to an assortment of biotic and abiotic stresses, particularly pathogen stress, which seriously affects their growth and development. In our investigation, MP infection gradually changed the expression of the sesame transcriptomes and demonstrated the most DEGs at 36 HPI, implying that it is the key period for sesame to resist the invasion of the pathogens. In addition, we found that DS had more DEGs and TFs than DR regardless of the time point post-inoculation, indicating that susceptible genotypes were more likely to be interfered by MP at the transcriptional level, which may be due to the lack of a corresponding mechanism in DS to adapt to MP stress. Different decisions made by DR and DS during pathogen infection may lead to their disparities in resistance.

Based on GO enrichment analysis at all four time points, a great deal of DEGs engaged in ribosome-related procedures were collected in DR specifically, however these DEGs were not found in DS, which indicated that pathogen infection seems to specifically trigger adapted transcription responses in DR. Ribosomes are ‘factories’ that synthesize proteins at the cellular level and various mechanisms have evolved to detect and react to environmental changes rapidly at transcriptional and translational levels in plants [23, 24]. More DEGs involved in ribosome-related pathways in DR demonstrates that DR might have a rapid and intense response to MP stress with translational mechanisms activating synergistically with that of transcription, which is consistent with the consequences described by Supriyo Chowdhury [25].

There were some DEGs constantly expressing in the two genotypes under MP stress, which represent the core genes mediating disease resistance against MP in sesame. It was discovered that many of these genes were PODs, and their expression increased significantly post-inoculation. Numerous studies have demonstrated that higher antioxidant enzyme activity helps to improve plant disease resistance [26, 27]. POD participates in the defence against pathogens through its role in the detoxification of H_2_O_2_ and it assumes an essential job in the process of disease resistance. When stressed by external pathogens, the enhancement of POD activity can increase the content of phenolic oxides to trigger hypersensitive responses and subsequently inhibit the proliferation and spread of pathogens [28].

There are many protein kinase genes and pathogenesis-related genes in this core gene set. It is realized that a significant number of receptor protein kinases and pathogenesis-related proteins can confer plant resistance against pathogens [29]. The PmDTM gene encoding receptor-like serine/threonine-protein kinase in wheat can improve the resistance of wheat to *Blumeria graminis f. sp. tritici* [30]. Similarly, the CsWAKL08 gene encoding a wall-associated receptor-like kinase was found to regulate resistance against *Xanthomonas citri subsp. citri* positively via a mechanism of ROS control and JA signalling, which further highlights the significance of this kinase family in plant disease resistance [31]. ScPR10 was identified as a pathogenesis-related gene from sugarcane, that positively regulates plant resistance against *Sporisorium scitamineum*, Sorghum mosaic virus, salicylic acid and methyl jasmonate stresses [32]. Another important gene family identified in this core gene set is cytochrome P450, one of the largest gene families in the plant genome. In wheat, the cytochrome P450 gene TaCYP72A was confirmed to confer resistance to deoxynivalenol, which mediated the early resistance of wheat to *F. graminearum* [33]. Likewise, Guilin Wang et al. discovered that the GbCYP86A1–1 gene in *Gossypium barbadense* plays a positive role in resistance against *Verticillium dahlia* and initiates the downstream immune pathways of disease resistance. For instance, GbCYP86A1–1 transgenic Arabidopsis significantly increased the expression of genes encoding protein kinases, TFs and PRs, thereby increasing its resistance [34]. Moreover, a few genes encoding laccase were also reflected in this gene set. Yan Zhang et al. reported that the GhLAC15 gene contained domains conserved by laccases enhances resistance against *Verticillium dahliae* by means of an increase in lignification and the accumulation of arabinose and xylose [35], which indicates that laccase may have a significant relationship with the resistance of plants to pathogens.

Furthermore, the gene expression of DR and DS during stress was compared, and 52 genes were screened as differentially and continuously expressed, indicating that they may provide DR with a higher resistance against charcoal rot. Accordingly, future research will focus on these genes involved in disease resistance in sesame and their biofunctions.

### PTI and ETI contribute to sesame resistance against MP

**Fig. 10:**
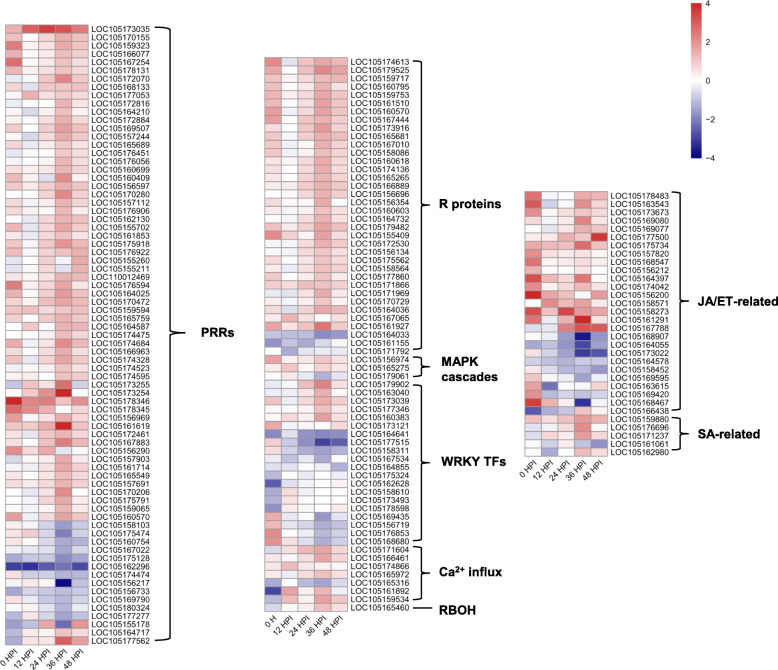
Heat maps of DEGs in plant immunity between DR and DS in sesame. Navy indicates down-regulated DEGs, and red indicates up-regulated DEGs in DR when compared to DS

To understand the mechanism of immune response of sesame to MP, 174 DEGs related to PTI and ETI were screened for following discussion through literatures, nr and KEGG annotation. These genes may be involved in the process of resistance of sesame to MP. They include 75 pattern recognition receptor (PRRs) genes, 36 resistance (R) genes, 1 respiratory burst oxidase homolog (RBOH), 7 Ca^2+^ influx related proteins, 3 MAPK cascades, 20 WRKY and 32 hormone metabolism related genes (Fig. 10, Additional file: Table S5).

### Pathogen perception and recognition by PRRs

The defence of plants begins with the detection of pathogen PAMP by PRR, then PRRs dynamically binds to different coreceptors, regulatory receptor kinases and receptor-like cytoplasmic kinases (RLCK) to initiate immune signalling transduction and PTI starts [2, 36]. Plant PRRs includes receptor-like kinase (RLK) and receptor-like protein (RLP) [37, 38]. There were 73 differentially expressed PRRs between the two genotypes involved in sesame resistance against MP. PRRs were induced in both genotypes, of which 12 were induced in DS and 60 in DR (Fig. 10, Additional file: Table S5). Previous studies have shown that some key PRRs in PTI such as brassinosteroid insensitive 1-associated kinase 1 (BAK1) [39], FLS2 [40] and chitin elicitor receptor kinase (CERK) [41], play an important role in plant resistance to pathogens. Notably, one CERK and two FLS2 genes exhibited differently between two genotypes. What’s more, WGCNA also showed that LRR-RLKs play an important role in sesame resistance to MP. It is implied that DR can identify PAMPs more rapidly and actively and induce PTI more strongly than DS, which may be the main reason for the difference in resistance between them.

### MAPK cascades, WRKY TFs, Ca^2+^ influx and RBOH

PRR-derived signals are transmitted by further phosphorylation cascades including MAPK cascades and calcium-dependent protein kinases (CDPKs) to the downstream targets such as the RBOH [42]. Similarly, in the process of sesame resistance to MP, there were three MAPKs differentially expressed, of which DR induced two (Fig. 10, Additional file: Table S5). WRKY TFs activated by MAPK cascades play a complex role in plant defence responses, which act as both positive [43, 44] and negative [45, 46] regulators. In this study, 20 WRKYs were differentially expressed in the two genotypes, of which 6 were induced in DR and 10 in DS (Fig. 10, Additional file: Table S5). It is unclear whether WRKY TFs play a positive or negative role in pathogen defence, which needs further study.

Furthermore, compared with DS, three calmodulin-like (CML) and three CDPKs were significantly up-regulated in DR post-inoculation (Fig. 10, Additional file: Table S5). When PRRs recognize the PAMPs of pathogens, a transient increasing of Ca^2+^ concentration can be observed in the cytoplasm. Ca^2+^ can bind to CML to induce downstream cell wall reinforcment and hypersensitive response, and it can also activate CDPK to phosphorylate and transduce immune signals such as RBOH proteins for downstream defence against pathogens [47]. It shows that Ca^2+^ is an important second messenger in sesame resistance to MP. One sesame gene (LOC105165460), the putative function of which is RBOH, was induced in DR. It is consistent with the observation of Fusarium wilt fungus pathogen in wheat, cotton and cucumber [48], indicating that there may be a higher level of ROS in DR post-inoculation, which inhibited the infection and colonization of MP.

### R proteins

Intracellular receptor R protein, an important component of plant immunity encoding by R gene, can detect and bind to pathogen effectors and trigger ETI, which has been provided strong evidences by previous researches. For example, R gene Pm60 mediates wheat resistance against powdery mildew [49], and two R genes RGA4 and RGA5 can interact each other functionally to mediate rice resistance to *Magnaporthe oryzae* [50]. In this study, a total of 36 R genes were differentially expressed between resistant and susceptible genotypes, of which 33 R genes were induced by DR (Fig. 10, Additional file: Table S5) and almost all R genes were differentially expressed at 36 HPI, which further indicated that 36 HPI was the key period. In addition, the up-regulated expression of R gene may represent the activation of ETI, indicating that DR induces a stronger ETI response than DS and enhances its resistance against MP.

### JA/ET and SA signalling pathways in sesame immunity

Phytohormones play an important role in the plant resistance to pathogens. It is believed that plant resistance to biotrophic pathogens is controlled largely by SA signalling pathways, while resistance to necrotrophic pathogens is mediated by the JA/ET signalling pathways [51]. It is found that MP, a hemi-biotrophic pathogen, has an obvious biological nutrition period of about 36 h and a transition period to necrotic nutrition, and then the necrotic nutrition stage in the interaction with sesame. The sesame resistance to MP is mainly caused by the activation of JA/ET signalling pathway [25]. Similarly, JA treatment or strong JA signalling pathway in strawberry fruits can enhance its resistance to *Botrytis cinerea*, indicating that JA was involved in grape resistance to *Botrytis cinerea* [52]. To study the potential role of JA/ET signalling pathway in this investigation, we detected the expression patterns of key DEGs involved in JA/ET signalling pathway and found that 17 DEGs were induced in DR while 5 were induced in DS. For instance, compared with DS, JA synthase genes (1 AOS, 1 LOX and 4 OPR) are up-regulated in DR. In addition, four JAZ proteins, one defensin and two MYC2 TFs were induced in DR (Fig. 10, Additional file: Table S5). Many literatures have shown that JA signalling pathway can directly regulate MYC2 TFs with JAZ proteins, and then downstream genes were induced [53]. In summary, JA signalling pathway may play an important role in sesame immunity. Compared with DS, there were 4 DEGs up-regulated while 4 DEGs down-regulated ET biosynthesis and signalling transduction pathway (Fig. 10, Additional file: Table S5), indicating that ET may not directly regulate the resistance against MP but play a dynamically regulated role in JA/ET signalling pathway.

**Fig. 11:**
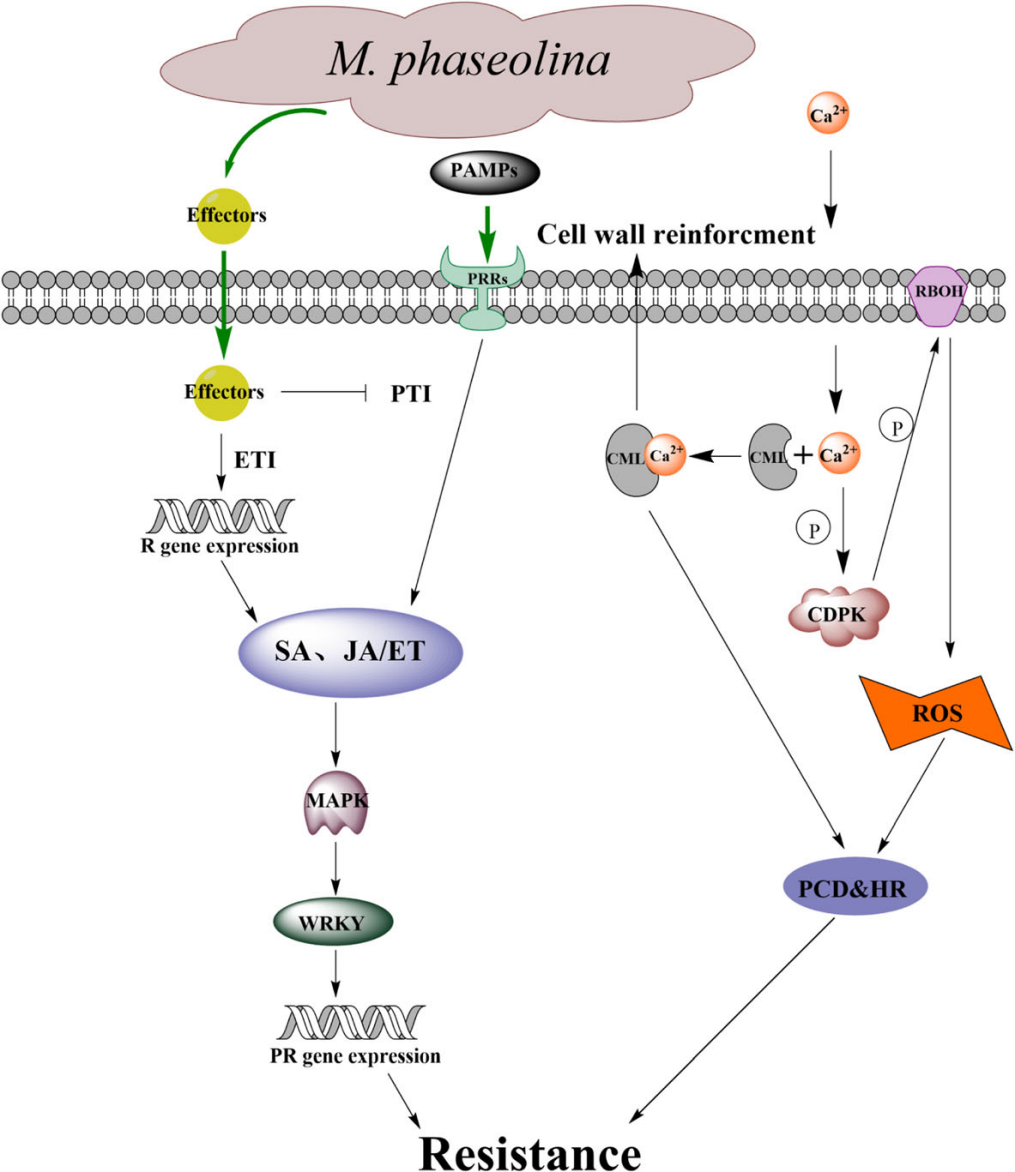
Diagram of putative major molecular reactions of the defensive responses to MP in DR

NPR1 protein is the master regulator induced by SA-mediated defence response, which is located in the downstream of SA signalling transduction and upstream of PR gene expression. NPR1, the positive regulatory gene in the systemic acquired resistance (SAR) pathway, can regulate the expression of PR proteins in SAR. For example, BjNPR1 transgenic mustard showed strong resistance against *Alternaria brassicae* and *Erysiphe cruciferarum*, and activated SAR, indicating that BjNPR1 was involved in the resistance to fungal pathogens [54]. In our research, three up-regulated genes encoding one NPR1 and two TGA TFs were detected in DR (Fig. 10, Additional file: Table S5). It has shown that the complex of TGA and NPR1 can enhance the binding of TGA and promoter of PR1 gene and induce the expression of PR1 actively [55]. In addition, one down-regulated gene encoding NIM1-INTERACTING2 (NIMIN2) was detected (Fig. 10, Additional file: Table S5). It is known that NIMIN2 can interact with NPR1, acting as its negative regulator, and repress the repression of NPR1-regulated genes [56]. All above showed that SA may assume a vital job in the sesame resistance to MP. It is shown that *T.long gibrachiatum* H9 can activate JA/ET and SA signalling pathways in cucumber, and then enhance the resistance to *Botrytis cinerea* [57]. Based on our results, a schematic illustration of the interactions between sesame and MP was shown as Fig. 11.

### Defence-related TFs

It has been reported that many TFs, such as bHLH [58, 59], MYB [60, 61], ERF [62–64], NAC [65, 66] and WRKY [43, 67, 68], are related to various plant resistance mechanisms against pathogens. In our study, the bHLH transcription factor family was the most abundant transcription factor in the interaction between sesame and MP. It is known that many members of the bHLH family are related to the abiotic stress resistance of plants, such as drought tolerance [69], cold tolerance [70] and salt tolerance [71]. However, their roles in plant biotic stress are rarely depicted. Qun Cheng et al. recently revealed the role of the bHLH transcription factor GmPIB1 in soybean phytophthora root rot and found that GmPIB1 can directly bind to the promoter of the key enzyme GmSPOD1, which encodes ROS and inhibits its expression, reducing the production of ROS and enhancing the resistance of soybean to Phytophthora [58]. Yan S et al. discovered that transgenic cucumber plants with the bHLH transcription factor CsIVP-RNAi had higher resistance to downy mildew and could accumulate higher levels of SA. CsIVP can physically interact with CsNIMIN1, a negative regulatory factor in SA signalling pathway, thus CsIVP is a significant regulatory factor in SA-mediated downy mildew resistance in cucumber [59]. Here, the current investigation also obtained a similar result: bHLH transcription factors may play an important role in sesame resistance (Fig. 7).

## Conclusions

In summary, a set of core genes, including protein kinases, disease-related proteins, cytochrome P450 and PODs and other genes closely related to disease resistance, were obtained in the sesame resistance to MP via comparative transcriptome analysis. Then, 52 genes expressed continuously and differentially between DR and DS were screened under MP press, which were enriched in GO terms such as response to abscisic acid, cell wall, hormone-mediated signalling pathway, response to hormone, cell-cell junction, defence response and signal transduction. Secondly, we preliminarily investigated the immune response mechanism of sesame against MP. Compared with DS, DR can respond to MP infection more quickly, with the translation mechanism and transcription mechanism activated cooperatively. DR is less likely to be interfered by MP at the transcriptional level. Furthermore, 174 genes involved in the PTI and ETI showed different expression patterns among resistant and susceptible genotypes, including PRRs and R genes that recognize pathogens, calcium ion influx process related and MAPK cascades related genes that phosphorylate and activate downstream signals, downstream genes such as RBOHs and genes related to hormone metabolism and transduction (JA/ET and SA). Of note, most of the PRRs, calcium ion influx related genes and R genes were induced in DR, indicating stronger PTI and ETI were triggered in DR. Finally, we found that JA/ET and SA signalling transduction pathway are both important in sesame resistance to MP. This is the first report on the mechanism of the interaction between sesame and MP using transcriptomic method, which provides more insights into the molecular mechanism of resistance genes in sesame against MP infection.

## Methods

### Materials and stress treatment

Two kinds of sesame accessions with contrasting levels of resistance disease-susceptible genotype (Ji 9014) and disease-resistant genotype (Zhengzhi No.13) [72, 73] used in this experiment were widely planted varieties and both of them were provided by Sesame Research Center, Henan Academy of Agricultural Sciences. *Macrophomina phaseolina* (MP), the pathogen of charcoal rot, was isolated and preserved by the Biocontrol Lab, Institute of Plant Protection, Henan Academy of Agricultural Sciences.

Preparation of stroma mixed with MP: The MP strain stored in 25% glycerol at − 20 °C was activated on PDA solid medium. After activation, MP was cultured in PDA solid medium and incubated in a 30 °C incubator for 4 days. The PDA culture medium full of MP was divided into pieces of approximately 0.5 cm with a sterilized toothpick, and then they were inoculated into the sterilized 200 mL liquid PD medium (each bottle was inoculated with half a plate of PDA). The medium was shaken and cultured for 5 days at 30 °C and 200 r/min. The mycelium suspension was obtained by breaking the culture medium full of mycelium with a tissue crusher. Then each 100 mL mycelium suspension was mixed with 100 mL sterilized water and 200 g sterilized stroma (nutritional soil: vermiculite = 3:1).

The sesame seedlings were cultured in the growth bowl of stroma (soil: nutritional soil: vermiculite = 3:1:1), thinning them in 2 pairs of true leaf stages, leaving 3 seedlings in each pot. They were cultured in an artificial climate box under the condition of 16 h light (30 °C) and 8 h darkness (28 °C). DS and DR were transplanted from the growth bowl to the stroma with mycelium at three pairs of true leaves. The root tissues of DS and DR were collected as samples (three biological replicates) for RNA extraction during MP treatment (12 h, 24 h, 36 h, 48 h) and before treatment (0 h).

### mRNA library construction and sequencing

Total RNA extracted with the TransZol Up Plus RNA Kit (Cat# ER501–01, Trans) was qualified by Agilent Bioanalyzer 2100 (Agilent Technologies, Santa Clara, CA, US) electrophoresis and purified with the RNA Clean XP Kit (Cat A63987, Beckman Coulter, Inc. Kraemer Boulevard Brea, CA, USA) and RNase-Free DNase Set (Cat#79254, QIAGEN, GmBH, Germany). The quality of the total RNA was checked by a NanoDrop ND-2000 spectrophotometer and an Agilent Bioanalyzer 2100 (Agilent Technologies, Santa Clara, CA, US), and the high-quality RNA after inspection could be sequenced later. According to the experimental operation instructions, the purified total RNA was subjected to mRNA separation, fragmentation, first-strand cDNA synthesis, second-strand cDNA synthesis, terminal repair, 3′- terminal addition of A, adapter junctions, enrichment and other steps to complete the construction of the cDNA library. After the construction of the library, a Qubit®2.0 Fluorometer was used to detect the concentration, and an Agilent 4200 was used to detect the size of the library.

Sequencing: According to the corresponding process shown by the cBot User Guide, cluster generation and first-direction sequencing primer hybridization were completed on the cBot equipped with an Illumina sequencer, and paired-end sequencing was carried out. The sequencing process and real-time data analysis were controlled by data collection software provided by Illumina.

### Transcriptome assembly

Before downstream analysis, unqualified reads with low quality, primer sequences and low terminal quality were removed. The Seqtk package was used to filter the raw reads to obtain clean reads so that the reads could be used for subsequent data analysis. After filtering, the clean reads were mapped to the sesame genome with HISAT2 (version:2.0.4) [74]. The data generated by mapping is a BAM file.

The sesame reference genome is S_indicum_v1.0 [22], which can be downloaded from: ftp://ftp.ncbi.nlm.nih.gov/genomes/all/GCF/000/512/975/GCF_000512975.1_S_indicum_v1.0/GCF_000512975.1_S_indicum_v1.0_genomic.fna.gz.

### Analysis of DEGs

Standardize gene expression by transforming reads into FPKM (fragments per kilobase of exon model per million mapped reads) to calculate gene expression level of each sample [75]. We first count the fragments number of each gene after Hisat2 alignment by Stringtie (version:1.3.0) [76, 77], then normalize it by TMM (trimmed mean of M values) method [78], and finally use perl script to calculate the FPKM value of each gene.

Differential genes between samples were analyzed by edgeR package [79], and the *p*-value was corrected by multiple hypothesis testing. The threshold of p-value was determined by controlling False Discovery Rate (FDR), and the corrected p-value was q-value [80, 81]. The differently expressed genes (DEG) were detected based on the parameters: log_2_ |Fold change| > =1 and q-value <= 0.05.

### Functional annotation and TFs prediction

DEGs were compared with the NCBI non-redundant (NR) database and were functionally annotated into GO and KEGG databases by KAAS.

To identify the transcription factors (TFs) in sesame DEGs, the online website plantTFDB [82] (http://planttfdb.cbi.pku.edu.cn/index.php?sp=Sin) was used.

### Weighted gene co-expression network analysis

Weighted gene co-expression network analysis (WGCNA) is a systems biology method used to describe gene correlation patterns among different samples. It can be used to identify gene sets with highly synergistic changes, and to analyze the interconnectedness of gene sets and the correlation between gene sets and phenotypes. WGCNA package version: 1.69 [83] in the R software was used to construct the gene co-expression networks in this research. After removing the inferior samples (meanFPKM < 0.1), the scale-free co-expression network was constructed by using the FPKM matrix transformed by log2, with the conditions that the mergeCutHeight was 0.8 and the minModuleSize was 30. To find out modules with biological significances, the correlation coefficient between eigengenes of modules and samples or sample traits was calculated. Cytoscape software version 3.6.1 [84] was used to perform visualization of each module with a cut off of the weight > 0.3 (obtained from the WGCNA).

### qRT-PCR

Relative expression levels of 15 genes in DS and DR quantified by the CFX 384™ real-time System made in Singapore and the 2× ChamQ Universal SYBR qPCR Master Mix (Vazyme, Nanjing, China) were calculated with the 2^-ΔΔCt^ method. Each sample had 3 replicates. The primers for qPCR were designed and synthesized in Sangon Biotech, they are shown in additional file: Table S4. The relative expression levels of 15 genes were normalized to that of the SiUBQ5 gene [85].

### Statistical analysis

All data in this study are the mean values of three biological replicates. The FPKM value was used to depict gene expression abundance.

## Supplementary Information

**Additional file 1: Table S1**. Genome mapping and gene mapping region.

**Additional file 2: Table S2**. DEGs involved in two vital pathways at 36 HPI in DS and DR.

**Additional file 3: Table S3**. Overall TFs in DR and DS.

**Additional file 4: Table S4**. Primer pair sequences for qPCR.

**Additional file 5: Table S5**. DEGs involved in the PTI and ETI in sesame resistance to MP.

**Additional file 6: Figure S1**. PCA of 30 samples.

**Additional file 7: Figure S2**. Overview of gene expression (FPKM> 0.1) in DS and DR.

**Additional file 8: Figure S3**. DEGs in DS and DR.

**Additional file 9: Figure S4**. Top 30 GO term enriched functional categories of DEGs up-regulated in DR at 12 HPI (A), 24 HPI (B), 36 HPI (C), 48 HPI (D).

**Additional file 10: Figure S5**. Top 30 GO terms enriched function categories of DEGs between DR and DS before-innoculation (0 h).

**Additional file 11: Figure S6**. Top 30 GO terms enriched function categories of 52 common DEGs between DR and DS.

## Data Availability

Data is available at NCBI SRA accession: PRJNA706471.

## Abbreviations

MP: *Macrophomina phaseolina*
DR: Disease-resistant
DS: Disease-susceptible
TF: Transcription factor
PTI: Pattern-triggered immunity
ETI: Effector-triggered immunity
PAMP: Pathogen-associated molecular pattern
ROS: Reactive oxygen species
MAPK: Mitogen activated protein kinase
SOD: Superoxidase dismutase
CAT: Catalase
POD: Peroxidase
bHLH: Basic helix-loop-helix
HPI: Hours post-inoculation
PCA: Principal component analyses
DEG: Differentially expressed genes
FDR: False discovery rate
PR1: Pathogenesis-related protein 1
HSP90: Heat shock protein 90 kDa beta
MAP 2 K1: Mitogen-activated protein kinase kinase 1
RPM1: Disease resistance protein RPM1
RBOH: Respiratory burst oxidase
FLS2: FLAGELLIN-SENSING 2
CDPK: Calcium-dependent protein kinase
BAK1: Brassinosteroid insensitive 1-associated receptor kinase 1
Pti1: Pto-interacting protein 1
MEKK1: Mitogen-activated protein kinase kinase kinase 1
NHO1: Glycerol kinase
RPS2: Disease resistance protein RPS2
AUX: Auxin
ABA: Abscisic acid
JA: Jasmonic acid
SA: Salicylic acid
ET: Ethylene
CRE1: Arabidopsis histidine kinase 2/3/4
B-ARR: Two-component response regulator ARR-B family
SnRK2: Serine/threonine-protein kinase SRK2
EIN2: Ethylene-insensitive protein 2
BZR1_2: Brassinosteroid resistant 1/2
BSK: BR-signalling kinase
CML: Calmodulin-like
GH3: Glycoside hydrolase
ARF: Auxin response factor
ERF1: Ethylene-responsive transcription factor 1
CYCD3: Cyclin D3
HR: Hypersensitive response
JAZ: Jasmonate ZIM domain-containing protein
WGCNA: Weighted gene co-expression network analysis
PRR: Ppattern recognition receptor
RLCK: Receptor-like cytoplasmic kinases
RLK: Receptor-like kinase
RLP: Receptor-like protein
CERK: Chitin elicitor receptor kinase
SAR: Systemic acquired resistance
NIMIN2: NIM1-INTERACTING2
PCD: Programmed cell death
FPKM: Fragments per kilobase of exon model per million mapped reads
