# Supplementary material for: The mechanism of sesame resistance against Macrophomina phaseolina was revealed via a comparison of transcriptomes of resistant and susceptible sesame genotypes
Source: BMC Plant Biol. 2021 Mar 29;21:159. doi: 10.1186/s12870-021-02927-5 (PMC8008628; doi:10.1186/s12870-021-02927-5)
Supplement: Supplementary file 5 — Additional file 5: Table S5. DEGs involved in the PTI and ETI in sesame resistance to MP. [file 12870_2021_2927_MOESM5_ESM.docx]

**Table S4.** DEGs involved in the PTI and ETI in sesame resistance to MP

| **Functional Category** | **Up/Down-regulated** | **Gene ID** | **Annotations** | **Log_2_ Fold Change** | | | | |
| --- | --- | --- | --- | --- | --- | --- | --- | --- |
|  |  |  |  | **0 HPI** | **12 HPI** | **24 HPI** | **36 HPI** | **48 HPI** |
| **PRRs** | **total of 75 DEGs** |  |  |  |  |  |  |  |
|  | **up** | LOC105173035 | Receptor serine/threonine kinase, RSTK | 1.44 | 2.91 | 3.53 | 3.18 | 2.46 |
|  |  | LOC105170155 | Receptor serine/threonine kinase, RSTK | 1.2 | — | — | 1.5 | 1.15 |
|  |  | LOC105159323 | Receptor serine/threonine kinase, RSTK | 2.41 | — | — | 1.55 | 1.07 |
|  |  | LOC105166077 | Receptor serine/threonine kinase, RSTK | 1.46 | — | — | 1.22 | — |
|  |  | LOC105167254 | Receptor serine/threonine kinase, RSTK | 2.65 | — | — | 1.13 | — |
|  |  | LOC105178131 | Receptor serine/threonine kinase, RSTK | 1.32 | — | — | 1.12 | — |
|  |  | LOC105172070 | Receptor serine/threonine kinase, RSTK | — | — | 1.02 | 2.21 | — |
|  |  | LOC105168133 | Receptor serine/threonine kinase, RSTK | — | — | 1.03 | 1.09 | 1.13 |
|  |  | LOC105177053 | Receptor serine/threonine kinase, RSTK | — | 1.45 | — | — | — |
|  |  | LOC105172816 | Receptor serine/threonine kinase, RSTK | — | — | — | 1.39 | — |
|  |  | LOC105164210 | Receptor serine/threonine kinase, RSTK | — | — | — | 1.19 | — |
|  |  | LOC105172884 | Receptor serine/threonine kinase, RSTK | — | — | — | 1.48 | — |
|  |  | LOC105169507 | Receptor serine/threonine kinase, RSTK | — | — | — | 1.74 | 1.18 |
|  |  | LOC105157244 | Receptor serine/threonine kinase, RSTK | — | — | — | 1.54 | 1.01 |
|  |  | LOC105165689 | Receptor serine/threonine kinase, RSTK | — | — | — | 1 | — |
|  |  | LOC105176451 | Receptor serine/threonine kinase, RSTK | — | — | — | 1 | — |
|  |  | LOC105176056 | Receptor serine/threonine kinase, RSTK | — | — | — | 1.35 | — |
|  |  | LOC105160699 | Receptor serine/threonine kinase, RSTK | — | — | — | 1.37 | — |
|  |  | LOC105160409 | Receptor serine/threonine kinase, RSTK | 1.25 | — | — | 2.09 | — |
|  |  | LOC105156597 | Receptor serine/threonine kinase, RSTK | 1.01 | — | — | 1.48 | 1.08 |
|  |  | LOC105170280 | Receptor serine/threonine kinase, RSTK | — | — | — | 2.19 | — |
|  |  | LOC105157112 | Receptor serine/threonine kinase, RSTK | — | — | — | 1.42 | — |
|  |  | LOC105176906 | Receptor serine/threonine kinase, RSTK | — | — | — | 1.34 | — |
|  |  | LOC105162130 | Receptor serine/threonine kinase, RSTK | — | — | — | 1.39 | 1.15 |
|  |  | LOC105155702 | Receptor serine/threonine kinase, RSTK | 1.36 | — | — | 1.68 | — |
|  |  | LOC105161853 | Receptor serine/threonine kinase, RSTK | — | — | — | 1.18 | — |
|  |  | LOC105175918 | Receptor serine/threonine kinase, RSTK | 1.03 | — | 1 | 1.98 | 1.32 |
|  |  | LOC105176922 | Receptor serine/threonine kinase, RSTK | 1.26 | — | — | 1.17 | 1.47 |
|  |  | LOC105155260 | Receptor serine/threonine kinase, RSTK | — | — | — | — | 1.59 |
|  |  | LOC105155211 | Receptor serine/threonine kinase, RSTK | — | — | — | — | 1.41 |
|  |  | LOC110012469 | Receptor serine/threonine kinase, RSTK | — | — | — | 1.44 | 1.32 |
|  |  | LOC105176594 | Receptor kinase, RK | 2.41 | — | — | 1.65 | — |
|  |  | LOC105164025 | Receptor kinase, RK | 1.43 | — | — | 1.86 | 1.16 |
|  |  | LOC105170472 | Receptor kinase, RK | — | — | 1.21 | 1.67 | 1.28 |
|  |  | LOC105159594 | Receptor kinase, RK | 1.09 | 1.14 | — | 1.02 | 1.2 |
|  |  | LOC105165759 | Receptor kinase, RK | — | — | — | 1.44 | 1 |
|  |  | LOC105164587 | Receptor kinase, RK | — | — | — | 1.39 | 1.37 |
|  |  | LOC105174475 | Receptor kinase, RK | — | — | — | 1.23 | — |
|  |  | LOC105174684 | Receptor kinase, RK | 1.51 | — | — | 2.12 | 1.34 |
|  |  | LOC105166963 | Receptor kinase, RK | — | — | — | 1.51 | — |
|  |  | LOC105174328 | Receptor kinase, RK | 1.1 | — | — | 1.67 | — |
|  |  | LOC105174523 | Receptor kinase, RK | — | — | — | 1.08 | — |
|  |  | LOC105174595 | Receptor-like protein, RLP | — | — | — | 1.18 | — |
|  |  | LOC105173255 | Receptor-like protein, RLP | — | 1.07 | — | 2.64 | — |
|  |  | LOC105173254 | Receptor-like protein, RLP | — | — | 2.04 | 4.09 | — |
|  |  | LOC105178346 | Receptor-like kinase, RLK | 4.4 | 2.27 | 2.38 | 1.1 | 2.04 |
|  |  | LOC105178345 | Receptor-like kinase, RLK | 2.76 | 2.09 | 1.46 | — | — |
|  |  | LOC105156969 | Receptor-like kinase, RLK | 1.84 | — | — | 1.53 | — |
|  |  | LOC105161619 | Receptor-like kinase, RLK | 1.03 | — | 1.52 | 5.18 | 1.12 |
|  |  | LOC105172461 | Receptor-like kinase, RLK | — | — | 1.05 | 1.37 | — |
|  |  | LOC105167883 | Receptor-like kinase, RLK | — | — | 1.15 | 2.38 | 1.32 |
|  |  | LOC105156290 | Receptor-like kinase, RLK | 2.1 | — | 1.6 | — | — |
|  |  | LOC105157903 | Receptor-like kinase, RLK | — | — | — | 1.58 | — |
|  |  | LOC105161714 | Receptor-like kinase, RLK | — | — | — | 1.6 | — |
|  |  | LOC105165549 | Receptor-like kinase, RLK | — | — | — | 1.27 | — |
|  |  | LOC105157691 | Receptor-like kinase, RLK | — | — | — | 1.4 | — |
|  |  | LOC105170206 | Receptor-like kinase, RLK | — | — | — | 2.14 | — |
|  |  | LOC105175791 | Receptor-like kinase, RLK | — | — | — | 1.58 | — |
|  |  | LOC105159065 | Receptor-like kinase, RLK | — | — | — | 1.38 | — |
|  |  | LOC105160570 | BRASSINOSTEROID INSENSITIVE 1-associated receptor kinase 1, BAK1 | 1.81 | — | — | 1.7 | 1.05 |
|  | **down** | LOC105158103 | Receptor serine/threonine kinase, RSTK | — | — | — | -1.55 | — |
|  |  | LOC105175474 | Receptor serine/threonine kinase, RSTK | — | — | — | -2 | — |
|  |  | LOC105160754 | Receptor serine/threonine kinase, RSTK | — | — | — | -1.31 | -1.21 |
|  |  | LOC105167022 | Receptor serine/threonine kinase, RSTK | — | — | — | -1.39 | — |
|  |  | LOC105175128 | Receptor serine/threonine kinase, RSTK | -1.33 | — | — | -1.81 | -1.4 |
|  |  | LOC105162296 | Receptor serine/threonine kinase, RSTK | -3.01 | -3.19 | -2.56 | -2.19 | -2.81 |
|  |  | LOC105174474 | Receptor kinase, RK | — | — | — | -1.15 | -1.13 |
|  |  | LOC105156217 | Receptor-like kinase, RLK | — | — | — | -3.83 | — |
|  |  | LOC105156733 | Receptor-like kinase, RLK | -1.37 | — | — | -1.03 | — |
|  |  | LOC105169790 | Receptor-like kinase, RLK | — | — | — | -1.11 | — |
|  |  | LOC105180324 | Receptor-like kinase, RLK | — | — | — | -1.15 | — |
|  |  | LOC105177277 | Receptor-like kinase, RLK | -1.11 | — | — | -1.4 | — |
|  | **mix** | LOC105155178 | Receptor serine/threonine kinase, RSTK | -1.51 | — | 1.64 | -2.3 | 1.95 |
|  |  | LOC105164717 | Receptor-like kinase, RLK | -1.07 | — | — | 1.12 | — |
|  |  | LOC105177562 | Receptor-like kinase, RLK | -1.28 | — | — | 2.92 | 1.79 |
| **R proteins** | **total of 36 DEGs** |  |  |  |  |  |  |  |
|  | **up** | LOC105174613 | leucine-rich repeat family protein | 2.16 | — | 1.12 | 1.63 | 1.33 |
|  |  | LOC105179525 | leucine-rich repeat family protein | 1.62 | — | 1.13 | 2.18 | 1.7 |
|  |  | LOC105159717 | leucine-rich repeat family protein | 1.12 | — | — | 1.24 | 1.33 |
|  |  | LOC105160795 | leucine-rich repeat family protein | 1.14 | — | — | 1.43 | — |
|  |  | LOC105159753 | leucine-rich repeat family protein | 1.36 | — | — | 1.61 | 1.04 |
|  |  | LOC105161510 | leucine-rich repeat family protein | 1.3 | — | — | 1.73 | 1.03 |
|  |  | LOC105160570 | leucine-rich repeat family protein | 1.81 | — | — | 1.7 | 1.05 |
|  |  | LOC105167444 | leucine-rich repeat family protein | 1.8 | — | 1.06 | 1.43 | — |
|  |  | LOC105173916 | leucine-rich repeat family protein | — | — | 1.04 | 1.85 | — |
|  |  | LOC105165681 | leucine-rich repeat family protein | — | — | — | 1.49 | 1.23 |
|  |  | LOC105167010 | leucine-rich repeat family protein | 1 | — | — | 1.47 | — |
|  |  | LOC105158086 | leucine-rich repeat family protein | 1.02 | — | — | 1.21 | — |
|  |  | LOC105160618 | leucine-rich repeat family protein | — | — | — | 1.56 | — |
|  |  | LOC105174136 | leucine-rich repeat family protein | — | — | — | 1.51 | — |
|  |  | LOC105165265 | leucine-rich repeat family protein | — | — | — | 1.39 | — |
|  |  | LOC105166889 | leucine-rich repeat family protein | — | — | — | 1.45 | 1 |
|  |  | LOC105156696 | leucine-rich repeat family protein | — | — | — | 1.44 | — |
|  |  | LOC105156354 | leucine-rich repeat family protein | — | — | — | 1.55 | — |
|  |  | LOC105160603 | leucine-rich repeat family protein | — | — | — | 1.16 | — |
|  |  | LOC105164732 | leucine-rich repeat family protein | — | — | — | 1.41 | — |
|  |  | LOC105179482 | leucine-rich repeat family protein | 1.13 | — | — | 1.7 | 1.19 |
|  |  | LOC105155409 | leucine-rich repeat family protein | 2.14 | — | — | 1.74 | — |
|  |  | LOC105172530 | leucine-rich repeat family protein | — | — | — | 1.47 | — |
|  |  | LOC105156134 | leucine-rich repeat family protein | — | — | — | 1.04 | — |
|  |  | LOC105175562 | leucine-rich repeat family protein | — | — | — | 1.08 | — |
|  |  | LOC105158564 | leucine-rich repeat family protein | — | — | — | 1.15 | — |
|  |  | LOC105177860 | leucine-rich repeat family protein | 1.01 | — | — | 1.07 | — |
|  |  | LOC105171866 | disease resistance protein | — | — | 1.21 | 1.12 | — |
|  |  | LOC105171969 | disease resistance protein | — | — | — | 1.57 | — |
|  |  | LOC105170729 | disease resistance protein | — | — | — | 1.04 | — |
|  |  | LOC105164036 | disease resistance protein | — | — | — | 1.58 | 1.3 |
|  |  | LOC105167065 | nematode resistance protein-like | — | 1.41 | — | — | — |
|  |  | LOC105161927 | plant cadmium resistance 2-like | 1.66 | — | 1.1 | 2.51 | — |
|  | **down** | LOC105164033 | disease resistance protein | — | -1.05 | -1.05 | -1.83 | -1.61 |
|  |  | LOC105161155 | disease resistance protein | -1.71 | — | -1.31 | — | — |
|  |  | LOC105171792 | disease resistance protein | — | -1.32 | — | — | — |
| **MAPK cascades** | **total of 3 DEGs** |  |  |  |  |  |  |  |
|  | **up** | LOC105156974 | mitogen-activated protein kinase kinase kinase | 1.71 | — | — | 1.03 | — |
|  |  | LOC105165275 | mitogen-activated protein kinase kinase kinase | — | 1.02 | — | — | — |
|  | **down** | LOC105179061 | mitogen-activated protein kinase | — | — | — | -1.22 | — |
| **WRKY TFs** | **total of 20 DEGs** |  |  |  |  |  |  |  |
|  | **up** | LOC105179902 | WRKY transcription factor, WRKY | — | — | 1.22 | 2.3 | — |
|  |  | LOC105163040 | WRKY transcription factor, WRKY | — | — | — | 1.48 | — |
|  |  | LOC105173039 | WRKY transcription factor, WRKY | 1.07 | — | — | 1.52 | — |
|  |  | LOC105177346 | WRKY transcription factor, WRKY | — | — | — | 1.17 | — |
|  |  | LOC105160383 | WRKY transcription factor, WRKY | — | — | — | 1.78 | — |
|  |  | LOC105173121 | WRKY transcription factor, WRKY | 1.59 | — | — | — | — |
|  | **down** | LOC105164641 | WRKY transcription factor, WRKY | -1.84 | — | -1.7 | -1.88 | -1.75 |
|  |  | LOC105177515 | WRKY transcription factor, WRKY | — | — | -1.77 | -2.94 | -2.53 |
|  |  | LOC105158311 | WRKY transcription factor, WRKY | — | — | -1.28 | -1.47 | -1.19 |
|  |  | LOC105167534 | WRKY transcription factor, WRKY | — | — | — | -1.31 | — |
|  |  | LOC105164855 | WRKY transcription factor, WRKY | — | — | — | — | -1.22 |
|  |  | LOC105175324 | WRKY transcription factor, WRKY | -1.1 | — | — | — | — |
|  |  | LOC105162628 | WRKY transcription factor, WRKY | -2.54 | — | — | — | — |
|  |  | LOC105158610 | WRKY transcription factor, WRKY | -1.29 | — | — | — | — |
|  |  | LOC105173493 | WRKY transcription factor, WRKY | -1.2 | — | — | — | — |
|  |  | LOC105178598 | WRKY transcription factor, WRKY | -1.74 | — | — | — | — |
|  | **mix** | LOC105169435 | WRKY transcription factor, WRKY | 1.5 | — | — | -1.64 | — |
|  |  | LOC105156719 | WRKY transcription factor, WRKY | 1.31 | — | — | -1.26 | — |
|  |  | LOC105176853 | WRKY transcription factor, WRKY | 2.1 | — | — | -1.09 | — |
|  |  | LOC105168680 | WRKY transcription factor, WRKY | 2.03 | — | — | -1.25 | — |
| **Ca2+ influx and RBOH** | **total of 8 DEGs** |  |  |  |  |  |  |  |
| **Ca2+ influx** |  |  |  |  |  |  |  |  |
|  | **up** | LOC105171604 | calmodulin-like | — | — | 1.33 | 1.66 | — |
|  |  | LOC105166461 | calmodulin-like | — | — | — | 1.66 | — |
|  |  | LOC105174866 | calcium-dependent protein kinase, CDPK | — | 1.23 | — | — | — |
|  |  | LOC105165972 | calcium-dependent protein kinase, CDPK | — | — | — | 1.16 | — |
|  | **down** | LOC105165316 | calmodulin-like | -1.33 | — | -1.09 | -1.41 | — |
|  | **mix** | LOC105161892 | calmodulin-like | -2.88 | 1.7 | — | 1.33 | — |
|  |  | LOC105159534 | calcium-dependent protein kinase, CDPK | -1.09 | 1.27 | — | 1.77 | 1.05 |
| **RBOH** |  |  |  |  |  |  |  |  |
|  | **up** | LOC105165460 | respiratory burst oxidase homolog protein | — | — | — | 1.19 | — |
| **Hormones** |  |  |  |  |  |  |  |  |
| **JA/ET** | **total of 27 DEGs** |  |  |  |  |  |  |  |
|  | **up** | LOC105178483 | allene oxide synthase, AOS | 2.62 | — | — | 1.51 | 1.33 |
|  |  | LOC105163543 | llipoxygenase, LOX | 3.05 | — | — | 2.08 | — |
|  |  | LOC105173673 | 12-oxophytodienoate reductase, OPR | 2.05 | — | — | 1.03 | — |
|  |  | LOC105169080 | 12-oxophytodienoate reductase, OPR | — | — | — | 2.24 | — |
|  |  | LOC105169077 | 12-oxophytodienoate reductase, OPR | — | — | — | 1.46 | 1.09 |
|  |  | LOC105177500 | 12-oxophytodienoate reductase, OPR | — | — | 1.49 | 1.02 | 3.69 |
|  |  | LOC105175734 | jasmonate ZIM-domain protein, JAZ | 1.54 | 1.3 | 1.19 | 2.7 | 1.14 |
|  |  | LOC105157820 | jasmonate ZIM-domain protein, JAZ | 2.05 | — | — | — | — |
|  |  | LOC105168547 | jasmonate ZIM-domain protein, JAZ | 2.6 | — | — | — | — |
|  |  | LOC105156212 | jasmonate ZIM-domain protein, JAZ | 1.41 | — | — | — | — |
|  |  | LOC105164397 | transcription factor MYC2, MYC2 | 3.22 | 1.32 | — | 2.54 | — |
|  |  | LOC105174042 | transcription factor MYC2, MYC2 | 2.14 | — | — | 1.29 | — |
|  |  | LOC105156200 | defensin | 4.72 | 1.63 | 1.14 | — | 1.85 |
|  |  | LOC105158571 | 1-aminocyclopropane-1-carboxylate synthase, ACS | — | 2.12 | 1.27 | 1.75 | — |
|  |  | LOC105158273 | 1-aminocyclopropane-1-carboxylate synthase, ACS | 3.24 | 1.14 | 3.39 | 1.88 | 1.68 |
|  |  | LOC105161291 | ethylene-responsive transcription factor 1, ERF1 | 2.58 | — | 1.44 | 4.08 | — |
|  |  | LOC105167788 | ethylene-responsive transcription factor 1, ERF1 | — | — | 2.36 | 3.12 | 3.02 |
|  | **down** | LOC105168907 | llipoxygenase, LOX | — | — | -1.56 | -3.62 | -1.65 |
|  |  | LOC105164055 | 1-aminocyclopropane-1-carboxylate synthase, ACS | -1.36 | — | -1.69 | -2.86 | -1.49 |
|  |  | LOC105173022 | 1-aminocyclopropane-1-carboxylate synthase, ACS | — | — | -1.45 | -3.05 | -2.67 |
|  |  | LOC105164578 | EIN3-binding F-box protein 1, EBF1 | — | — | -1.17 | — | — |
|  |  | LOC105158452 | ethylene-responsive transcription factor 1, ERF1 | — | — | -1.12 | -1.08 | -1.63 |
|  | **mix** | LOC105169595 | allene oxide synthase, AOS | 1.07 | — | — | -1.44 | — |
|  |  | LOC105163615 | llipoxygenase, LOX | 1.74 | -2.21 | — | — | — |
|  |  | LOC105169420 | 12-oxophytodienoate reductase, OPR | 2.04 | -1.09 | — | — | — |
|  |  | LOC105168467 | jasmonate ZIM-domain protein, JAZ | 3.51 | 1.4 | — | -3.2 | — |
|  |  | LOC105166438 | defensin | -2.59 | -1.26 | — | 1.08 | — |
| **SA** | **total of 5 DEGs** |  |  |  |  |  |  |  |
|  | **up** | LOC105159880 | NPR1 | 1.14 | — | 1.16 | 1.87 | 1.42 |
|  |  | LOC105176696 | transcription factor TGA | — | — | — | 2.09 | — |
|  |  | LOC105171237 | transcription factor TGA | — | — | — | 1.8 | — |
|  | **down** | LOC105161061 | protein NIM1-INTERACTING 2, NIMIN-2 | — | — | — | — | -1.74 |
|  | **mix** | LOC105162980 | chitinase | — | -1.31 | — | 1.27 | — |
