# Supplementary figures and images for: The mechanism of sesame resistance against Macrophomina phaseolina was revealed via a comparison of transcriptomes of resistant and susceptible sesame genotypes
Source: BMC Plant Biol. 2021 Mar 29;21:159. doi: 10.1186/s12870-021-02927-5 (PMC8008628; doi:10.1186/s12870-021-02927-5)

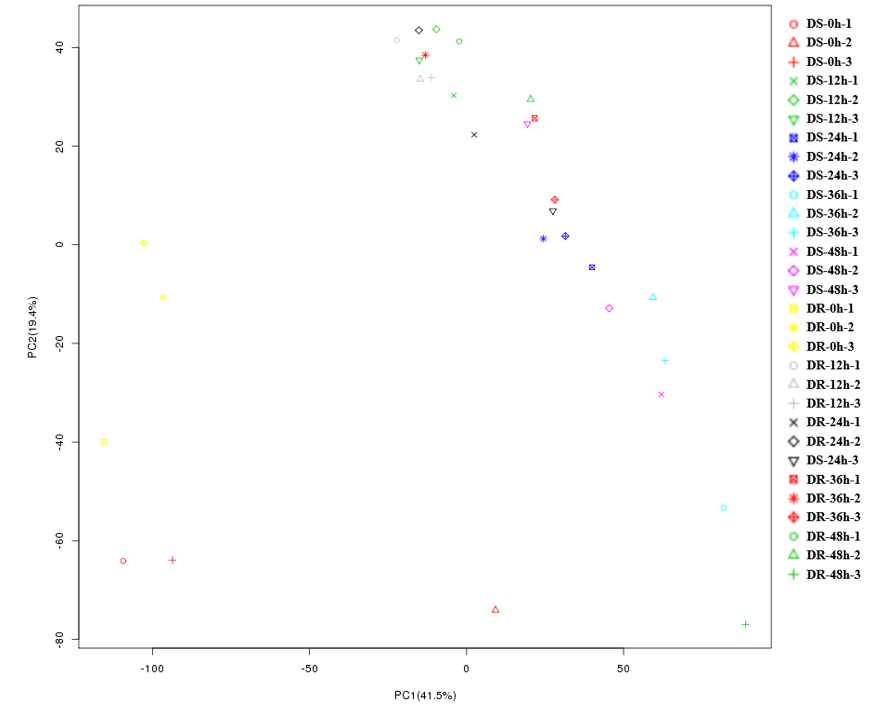


**Figure S1.** PCA of 30 samples.

Supplement: Supplementary file 6 — Additional file 6: Figure S1. PCA of 30 samples. [file 12870_2021_2927_MOESM6_ESM.docx]

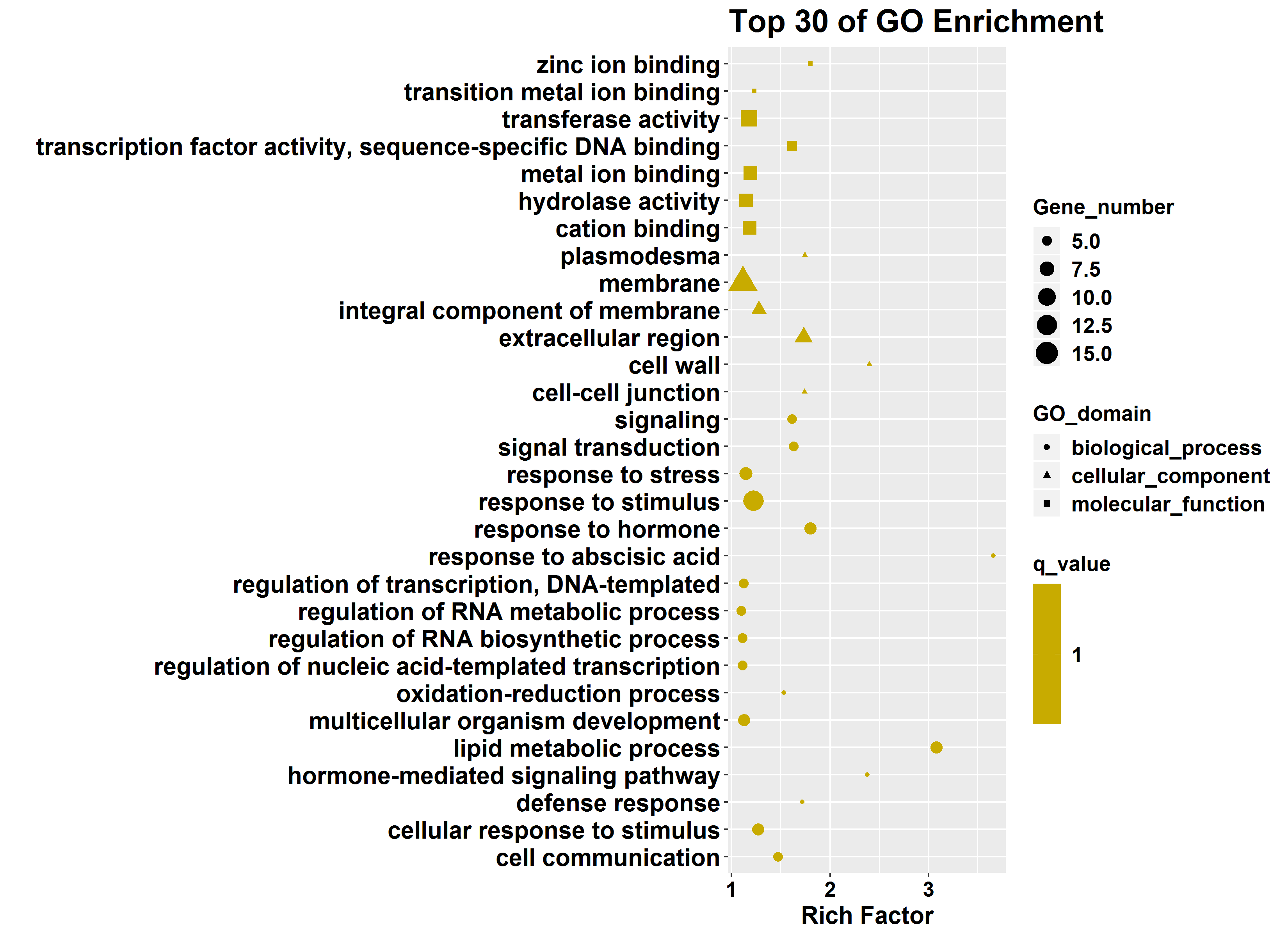


**Figure S6.** Top 30 GO terms enriched function categories of 52 common DEGs between DR and DS.

Supplement: Supplementary file 11 — Additional file 11: Figure S6. Top 30 GO terms enriched function categories of 52 common DEGs between DR and DS. [file 12870_2021_2927_MOESM11_ESM.docx]
