# Supplementary material for: The mechanism of sesame resistance against Macrophomina phaseolina was revealed via a comparison of transcriptomes of resistant and susceptible sesame genotypes
Source: BMC Plant Biol. 2021 Mar 29;21:159. doi: 10.1186/s12870-021-02927-5 (PMC8008628; doi:10.1186/s12870-021-02927-5)
Supplement: Supplementary file 7 — Additional file 7: Figure S2. Overview of gene expression (FPKM> 0.1) in DS and DR. [file 12870_2021_2927_MOESM7_ESM.docx]

**Figure S1.** PCA of 30 samples.


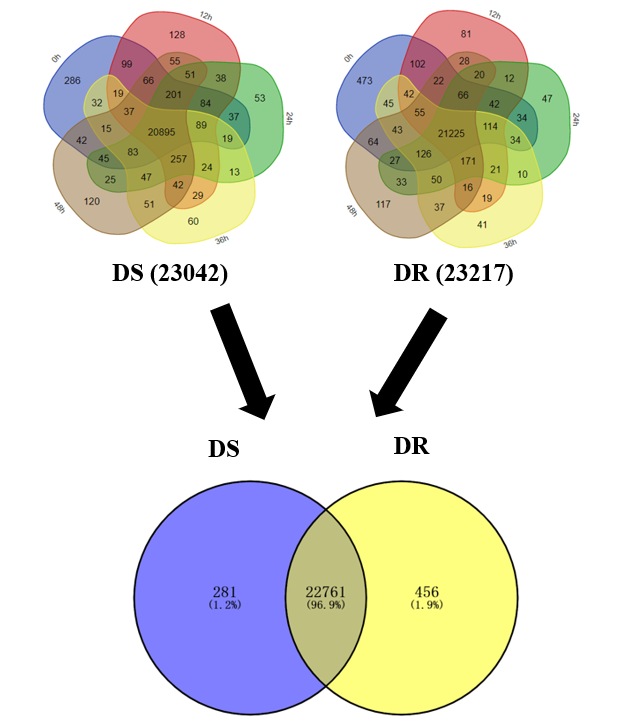


**B**

**A**

**Figure S2.** Overview of gene expression (FPKM>0.1) in DS and DR.

**A**. Gene numbers expressed at five time points post-inoculation.

**B.** Overall gene numbers expressed in DS and DR
