# Supplementary material for: The mechanism of sesame resistance against Macrophomina phaseolina was revealed via a comparison of transcriptomes of resistant and susceptible sesame genotypes
Source: BMC Plant Biol. 2021 Mar 29;21:159. doi: 10.1186/s12870-021-02927-5 (PMC8008628; doi:10.1186/s12870-021-02927-5)
Supplement: Supplementary file 8 — Additional file 8: Figure S3. DEGs in DS and DR. [file 12870_2021_2927_MOESM8_ESM.docx]

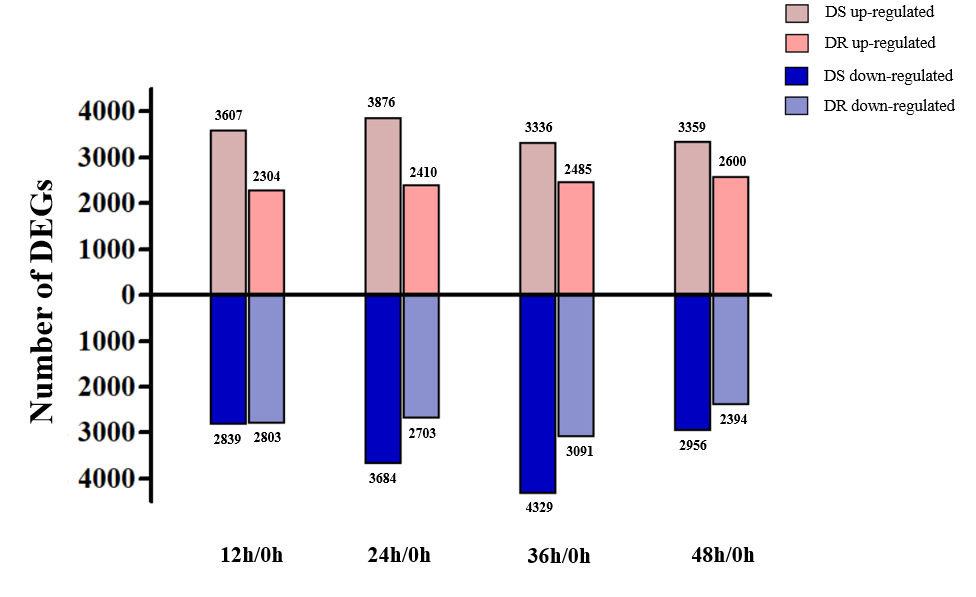

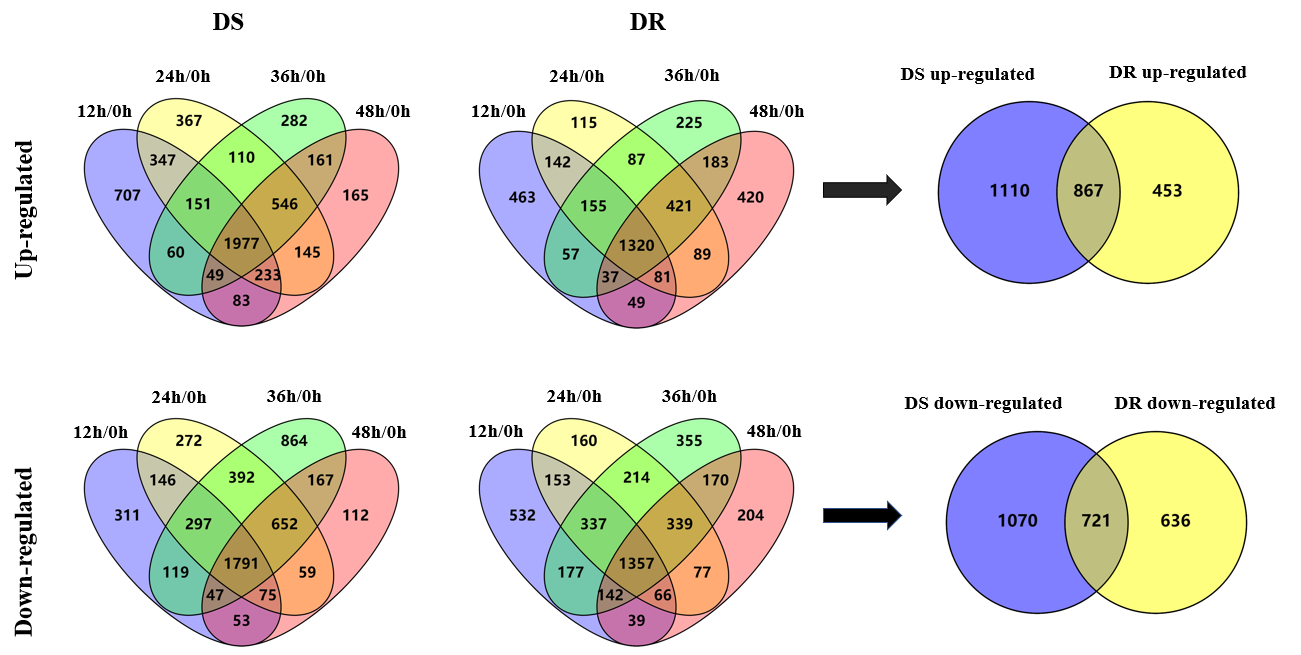


**A**

**B**

**Figure S3.** DEGs in DS and DR.

**A.** DEG numbers in DS and DR at four time points post-inoculation.

**B.** Up- and down-regulated DEGs compared between DS and DR.
