# Supplementary material for: The mechanism of sesame resistance against Macrophomina phaseolina was revealed via a comparison of transcriptomes of resistant and susceptible sesame genotypes
Source: BMC Plant Biol. 2021 Mar 29;21:159. doi: 10.1186/s12870-021-02927-5 (PMC8008628; doi:10.1186/s12870-021-02927-5)
Supplement: Supplementary file 9 — Additional file 9: Figure S4. Top 30 GO term enriched functional categories of DEGs up-regulated in DR at 12 HPI (A), 24 HPI (B), 36 HPI (C), 48 HPI (D). [file 12870_2021_2927_MOESM9_ESM.docx]

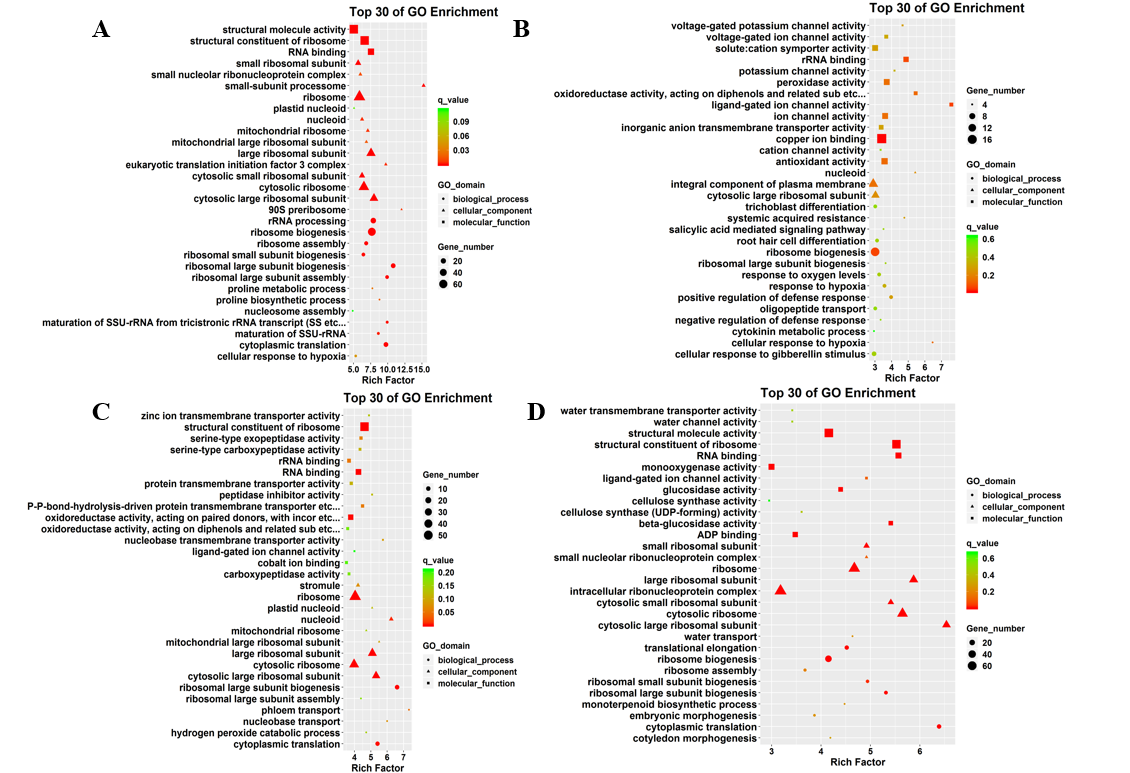


**Figure S4.** Top 30 GO term enriched functional categories of DEGs up-regulated in DR at 12 HPI (A), 24 HPI (B), 36 HPI (C), 48 HPI (D).
