# Supplementary material for: The mechanism of sesame resistance against Macrophomina phaseolina was revealed via a comparison of transcriptomes of resistant and susceptible sesame genotypes
Source: BMC Plant Biol. 2021 Mar 29;21:159. doi: 10.1186/s12870-021-02927-5 (PMC8008628; doi:10.1186/s12870-021-02927-5)
Supplement: Supplementary file 10 — Additional file 10: Figure S5. Top 30 GO terms enriched function categories of DEGs between DR and DS before-innoculation (0 h). [file 12870_2021_2927_MOESM10_ESM.docx]

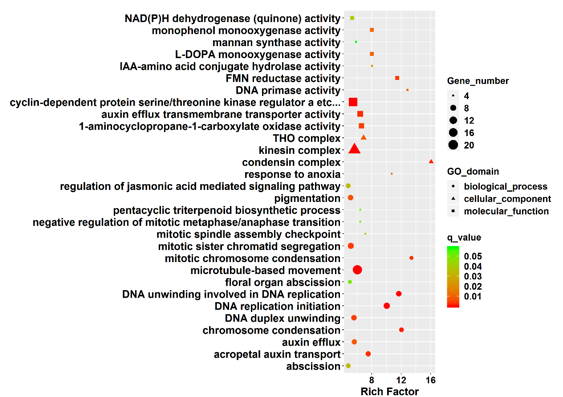


**Figure S5.** Top 30 GO terms enriched function categories of DEGs between DR and DS before-innoculation (0h).
